# Ion regulatory mechanisms supporting hypersaline tolerance in the coastal rock pool mosquito *Aedes togoi*

**DOI:** 10.1016/j.isci.2026.118153

**Published:** 2026-11-07

**Authors:** Jonathan Chiang, Elissa Khodikian, Orna Phelan, Ana K. Parra, Daniel A.H. Peach, Andrea C. Durant, Benjamin J. Matthews

**Affiliations:** 1 The University of British Columbia, Department of Zoology, Vancouver, BC V6T1Z4, Canada; 2 University of Washington, Department of Biology, Seattle, WA 98195, USA; 3 University of Georgia, Savannah River Ecology Laboratory, Aiken, SC 29802, USA; 4 University of Georgia, Department of Infectious Diseases, Athens, GA 30602, USA

**Keywords:** ion regulation, saline tolerance, osmoregulation, Aedes togoi, mosquito genomics

## Abstract

The coastal rock pool mosquito, *Aedes togoi,* is a remarkable example of a saline-tolerant mosquito. While most mosquitoes are obligate freshwater breeders, *Ae. togoi* larvae and pupae inhabit coastal rock pools that span freshwater to hypersaline conditions. We explore the ability of *Ae. togoi* larvae to tolerate hypersaline conditions by profiling the salt gland, an ion regulatory nexus within an anatomically unique, elaborated anal canal. We present a *de-novo* chromosome-scale genome assembly for *Ae. togoi*, highlighting its relatively small genome size and differences in chromosomal structure as compared to other *Aedine* mosquitoes. We detail repeat content and curate key multi-gene families encoding ion-transport proteins enriched in the larval salt gland, building a model of the machinery that likely facilitates hyperosmotic urine formation. Our work provides insights into molecular and anatomical adaptations that underpin the ability of mosquitoes and other insects to thrive in hypersaline rock pools and other dynamic environments.

## Highlights

• The coastal rock pool mosquito *Aedes togoi* tolerates a wide range of salinity
• *Aedes togoi* has a compact genome compared to other Aedine mosquitoes
• Ion transport gene expression is enriched in an anatomically specialized salt gland
• Salt gland transport machinery likely drives hyperosmotic urine formation

## Graphical abstract

**Figure:**
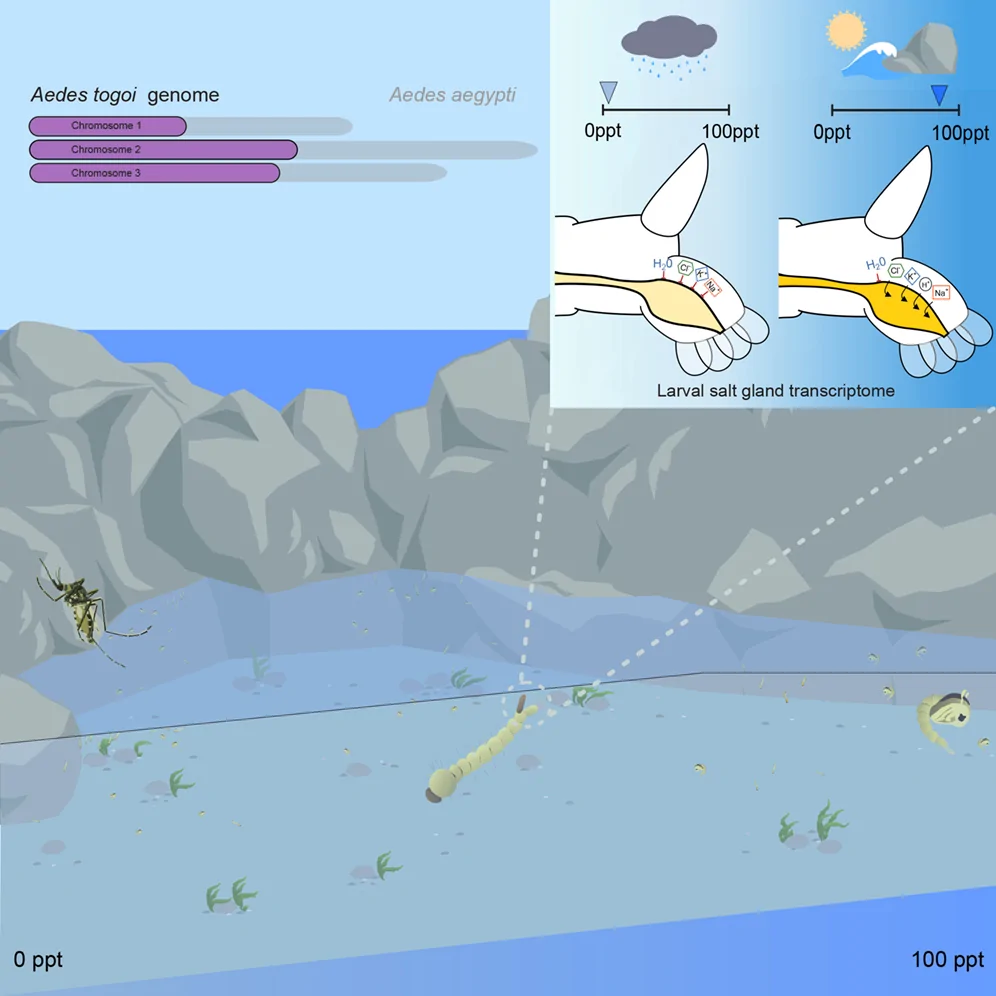

## Teaser

Biological sciences; Bioinformatics; Aquaculture

## Introduction

Only a small subset of the >3500 extant species of mosquitoes are saline-tolerant.^1^^,^^2^ Aquatic larvae of these saline-tolerant species develop and thrive in stagnant saline waters such as coastal rock pools or tidal marshes, while also contending with rapid dilution of the water from rainfall or concentration of salt from evaporation; in other words, they are euryhaline. Larvae thrive in these fluctuating habitats owing to a suite of anatomical and physiological innovations; however, the genetic underpinnings of mosquito saline tolerance are almost entirely unknown.

The larvae of saline-tolerant mosquito species are remarkable in their ability to develop across salinity extremes. *Ae. togoi* is found along the Northern Pacific coastline of North America and in coastal regions of East Asia, spanning subtropical to subarctic latitudes, where it is a potential vector for flaviviruses and filarial parasites.^3^^,^^4^^,^^5^ Like other mosquito species, *Ae. togoi* requires a suitable water source to continue its life cycle: a place to lay its eggs and for its offspring to develop in. Larvae of most mosquito species are restricted to development in clean freshwater; however, *Ae. togoi* are among the estimated 5% of euryhaline species that can survive and develop in freshwater, brackish, and saline waters.^1^^,^^6^^,^^7^^,^^8^^,^^9^^,^^10^ Inhabiting coastline regions may have been an adaptation to occupy an ecological niche with less competition from freshwater-obligate species.^11^^,^^12^ The coastal rock pools that *Ae. togoi* inhabit experience wide-ranging fluctuations in environmental conditions, most notably temperature and salinity, as they are generally unshaded and filled by both salt spray and rainfall.

In freshwater, hypo-osmotic conditions pose an osmoregulatory need for aquatic animals to counteract passive ion loss and water gain. By contrast, in hyper-osmotic conditions, such as in seawater, there is an increased risk of desiccation to the ion-concentrated surrounding environment. Euryhaline mosquito larvae that develop in coastal habitats, including *Ae. togoi*, regularly experience rapid changes in water salinities ranging from dilute to hypersaline, several times more concentrated than seawater.^6^^,^^13^ The physiological feats of euryhaline mosquito larvae during their development in these habitats of salinity extremes are impressive; a larva maintains a near-constant hemolymph osmotic pressure by hyper-regulating in dilute freshwater by actively absorbing ions and hypo-regulating in saline water by actively excreting ions.^1^^,^^6^^,^^14^^,^^15^ There is some information from saline-tolerant species spanning mosquito genera about the physiological mechanisms that lead to salt and water balance^1^^,^^16^^,^^17^ but these remain unresolved for *Ae. togoi*.

In other euryhaline mosquitoes that routinely develop across salinity extremes, saline conditions have selected for anatomical modifications of the larval hindgut that involve specialized segments that actively secrete salts and concentrate the urine. Perhaps the best characterized examples of these salt-secreting organs are the posterior rectal segments of the two-part rectum of Aedine saline-tolerant larvae, *Aedes taeniorhynchus* and *Aedes campestris*,^16^^,^^17^ and the specialized dorsal anterior rectal (DAR) cells in two Anopheles species, *Anopheles gambiae* and *Anopheles albimanus*.^18^ Additionally, previous characterization of proteins involved in ion regulation in euryhaline species has documented how their expression and localization within the salt-secreting tissues change in response to fluctuations in osmotic pressure and osmoregulatory needs as ambient salinity changes.^16^^,^^18^ Instead of a specialized segment being situated near or within the rectum, the salt-secreting organ of *Ae. togoi larvae* has emerged further posteriorly along the hindgut, as an elaboration of part of the anal canal.^6^^,^^19^

Knowledge of the ion-transport machinery that drives the production of hyperosmotic urine via the salt gland of euryhaline larvae is very much in its infancy. However, recent work on euryhaline *Ae. taeniorhynchus* larvae demonstrate that active sodium secretion is powered by the V-type H^+^-ATPase.^16^ Does the salt gland of *Ae. togoi* larvae employ active sodium secretion when larvae are developing in high-salinity water? To address this question and other aspects of *Ae. togoi’s* unique biology, we present a *de novo* chromosome-scale genome assembly and accompanying protein-coding gene-set annotations. Furthermore, we use a bulk RNA-sequencing approach from both whole larval tissues and salt gland tissues from animals reared in freshwater (FW) or seawater (SW) to begin to resolve ion regulatory pathways and highlight candidate transcripts that are potentially important to these processes. Together, these data and resources contribute useful reference points to support future comparative physiology and evolutionary genomics studies, provide key insight into osmoregulation in euryhaline mosquitoes, and lay a solid foundation for further physiological studies in *Ae. togoi*.

Recent advances in long-read sequencing and chromatin conformation capture technologies have revolutionized our ability to study mosquito genomes in their full complexity. High-quality, chromosome-scale assemblies for various mosquito species have revealed striking differences in repetitive element content, genome organization, and chromosome structure, providing insights into mosquito evolution.^20^^,^^21^^,^^22^^,^^23^^,^^24^^,^^25^^,^^26^^,^^27^ These resources for vector and non-vector species alike have paved the way for fundamental discoveries into many processes of mosquito behavior and physiology, with potential implications for disease control, including characterization of the genes and molecular mechanisms underpinning vector competence, vector transmission, and environmental adaptation. Yet, genetic resources that make such discoveries possible are still sparse, particularly among saline-tolerant mosquitoes.^28^^,^^29^^,^^30^^,^^31^^,^^32^^,^^33^^,^^34^^,^^35^ Within *Aedes* mosquitoes to date, there are only two publicly available genome assemblies from saline-tolerant species (*Ae. camptorhynchus*, GCF_037179485.1; *Ae. polynesiensis*, GCA_051529985.1), only one of them with gene annotations.

Here, we advance the availability of genetic resources for the study of mosquito saline tolerance, including the very first chromosome-scale genome assembly of a saline-tolerant *Aedes* mosquito (the coastal rock pool mosquito, *Ae. togoi*) and a transcriptome of a novel salt-secreting organ found in some saline-tolerant mosquito larvae, and build toward understanding the genetic underpinnings of mosquito saline tolerance.

## Results

### Generation of an *Ae. togoi* colony from British Columbia, Canada

To facilitate the generation of a reference genome for *Ae. togoi*, we first established a laboratory colony from field collections in Lighthouse Park, West Vancouver, British Columbia, Canada (Figure 1). In addition to collecting larvae and pupae to establish a laboratory colony, we recorded water temperature, salinity, and larval and pupal abundance from coastal rock pools over the course of a year to define the conditions that this population of *Ae. togoi* might routinely encounter and tolerate in the field (Table S1). The large majority of *Ae. togoi* larvae and pupae were found during the summer months (May-September), when water temperature and salinity were higher (Table S1). These data show that larvae and pupae from this population of *Ae. togoi* can be found in water temperatures from ∼1.5°C to 30 °C and salinity from 0 to 84.8 ppt (Figure 1B) before emerging as adults (Figure 1C).

**Figure 1:**
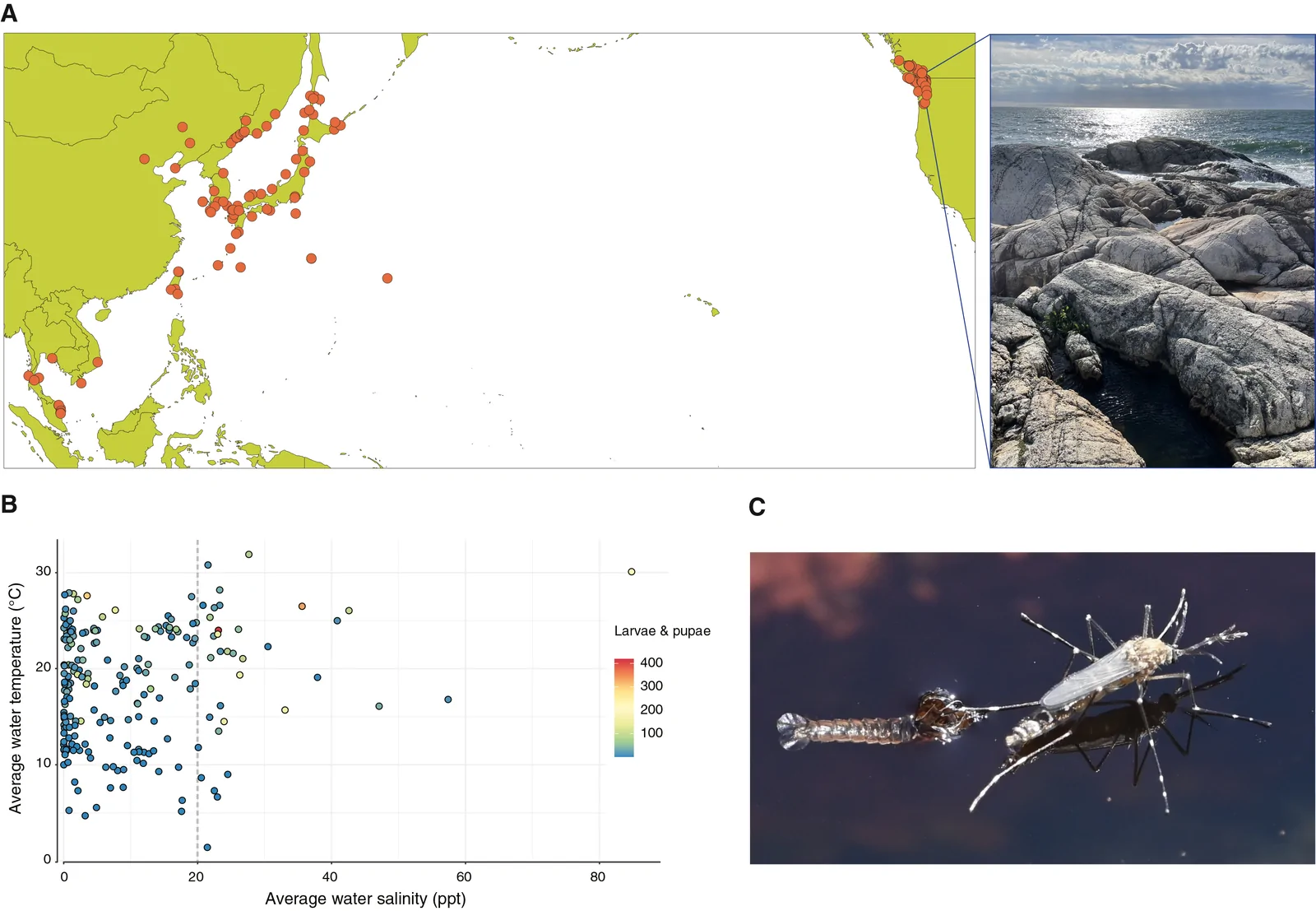
Salinity tolerance and global distribution of *Aedes togoi* (A) Global distribution of *Ae. togoi*, with locations where *Ae. togoi* are present indicated in orange. Observation data re-plotted from Peach and Matthews, 2020. Right image shows *Ae. togoi* coastal rockpool habitat in Lighthouse Park in West Vancouver, Canada. (B) Water temperature (C) and salinity (ppt) of coastal rockpools with *Ae. togoi* larvae or pupae present were measured over the course of a year to capture the range of conditions that *Ae. togoi* are exposed to. The dotted line at 20 ppt represents the average surface water salinity of seawater in the area surrounding the sampling site. Color represents the number of *Ae. togoi* larvae and pupae sampled in a rockpool on a single day. (C) Adult *Ae. togoi* newly emerged from a coastal rockpool.

### Establishment of *Ae. togoi* genomic resources through DNA and RNA sequencing

As the basis for a *de novo* reference genome assembly, we extracted high molecular weight (HMW) genomic DNA from *Ae. togoi* pupae taken from our laboratory colony. HMW DNA collected from a single pupa was used to generate 4.25 million Q20 PacBio HiFi reads across two flow cells, with a combined mean read length of 13.4 kb, representing 56.8 Gbp of total sequencing. From another single pupa, Hi-C data were collected through crosslinking, digestion, ligation, and sequencing, resulting in 68.3 Gbp of sequence across 424.6 million paired-end Illumina reads. Additionally, 17.5 Gbp across 2.5 million Nanopore (ONT) reads were generated from a single presumptive male pupa, 12.2 Gbp across 2.1 million ONT reads were generated from a single presumptive female pupa, 23.1 Gbp across 2.1 million ONT reads were generated from a pool of eight presumptive male pupae, and 20.4 Gbp across 1.6 million ONT reads were collected from a pool of eight presumptive female pupae (all reads deposited at SRA, details within Table S2). Pupae used in sequencing were collected for DNA extraction 1–2 generations (PacBio), 15–16 generations (Hi-C), or 24–25 generations (ONT) after colony establishment and, in the case of ONT sequencing, were determined to be likely male or female based on pupal morphology.

To facilitate the annotation of our genome, mRNA-sequencing datasets were collected from various tissues and life stages. mRNA collected from *Ae. togoi* adults (Figure 2A) generated 1,680.7 million raw reads from pooled head libraries and 1,603.0 million raw reads from pooled body libraries (Table S2). Additionally, mRNA extracted from pooled whole larvae generated 182.3 million raw reads and mRNA from pooled larval salt glands generated 694.9 million raw reads (Table S2). Adults and larvae used in sequencing were collected for RNA extraction 24–30 generations after colony establishment.

**Figure 2:**
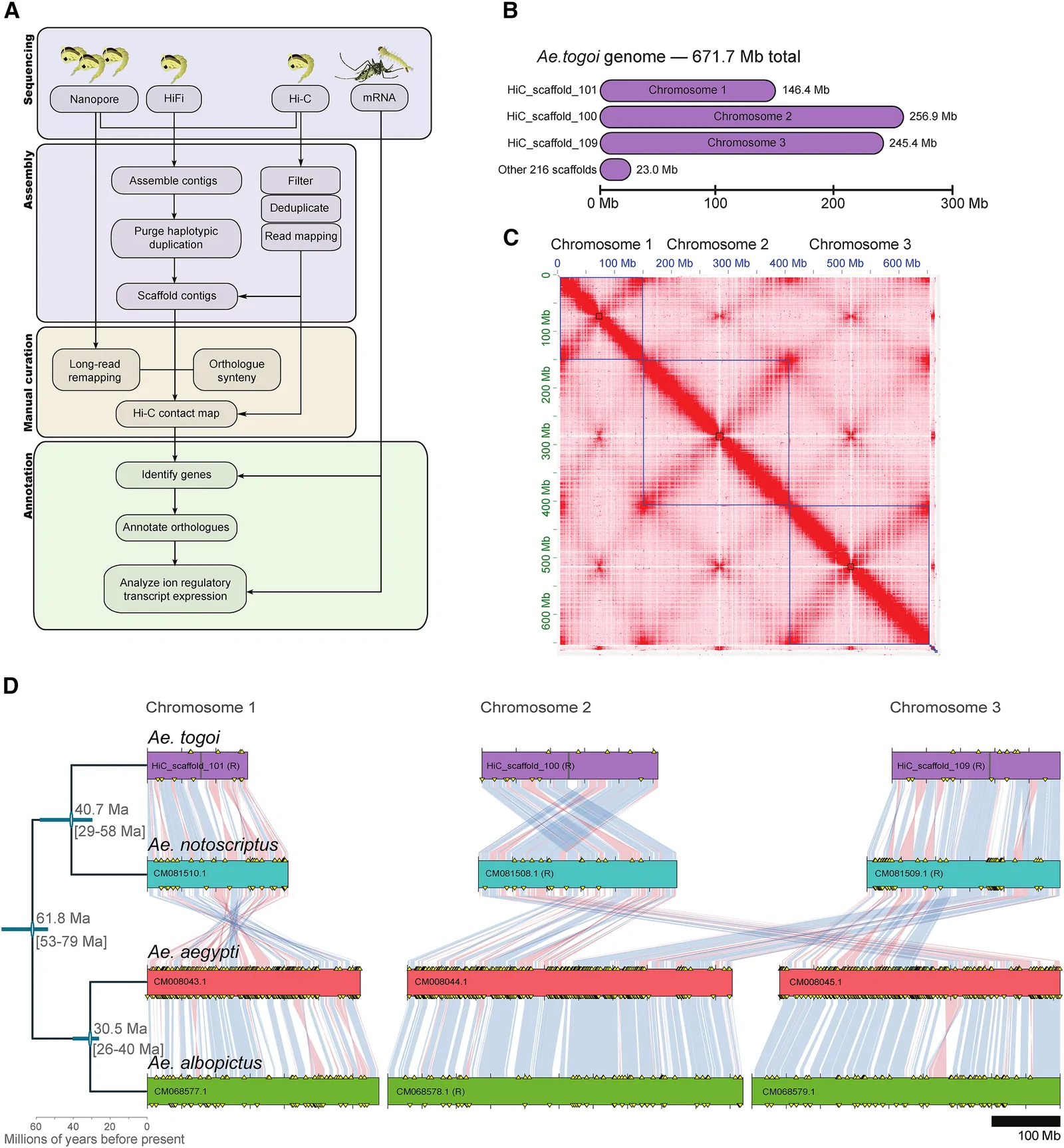
Genome assembly and chromosome structure of the saline-tolerant *Aedes togoi* (A) Overview of genomic and transcriptomic sequencing data collection, genome assembly steps, and gene annotation strategy. (B) Scaffold and full genome lengths for the chromosome-scale *Ae. togoi* assembly. (C) Hi-C contact map highlighting chromosome structure of the curated *Ae. togoi* genome assembly. Presumed centromere regions are approximately labeled with black borders. (D) Chromosomal synteny between *Ae. togoi*, *Ae. notoscriptus*, *Ae.aegypti*, and *Ae. albopictus* genomes based on the location of orthologous BUSCO genes. Presumed centromere regions on Ae. togoi chromosomes are shown as gray bands. Chromosomal inversions are highlighted in red while syntenic blocks with matching orientation are denoted in blue. Chromosomes with the majority of synteny on the opposite strand are labeled with (R). Divergence times of the comparison species are median values with 95% credible intervals in square brackets, taken across 100 pruned posterior trees from Soghigian et al. (2023).

### Genome assembly and completeness metrics

The initial assembly of HiFi reads, along with Hi-C and ultra-long ONT read integration, resulted in a primary contig assembly with 849 contigs and a total contig sequence length of 700.438 Mb (Table 1). Additionally, haplotype-phased contig-level assemblies were generated: a haplotype_1 assembly with 1220 contigs and a total contig sequence length of 630.117 Mb, and a haplotype_2 assembly with 1153 contigs and a total contig sequence length of 680.568 Mb. After purging haplotypic duplication from the primary contig assembly, the assembly consisted of 749 contigs and had a total contig sequence length of 671.670 Mb (Table 1). Following Hi-C-aware scaffolding, the assembly was composed of 212 scaffolds and had a total scaffold sequence length of 671.726 Mb (Table 1). Following manual curation, the assembly had 219 scaffolds, including the three chromosome-scale scaffolds expected of mosquitoes, and a total scaffold sequence length of 671.695 Mb; after gap-closing, the assembly had 219 scaffolds and a total scaffold sequence length of 671.687 Mb (Figures 2A–2C; Table 1).

**Table 1:** Statistics of assembly at different stages

Assembly stage	Length	Number of contigs	Contig N50	Number of scaffolds	Scaffold N50	GC content	BUSCO completeness
Contig assembly	700.438 Mbp	849	2.784 Mbp	N/A	N/A	39.07%	96.90%
Haplotypic duplication purged	671.670 Mbp	749	2.835 Mbp	N/A	N/A	39.06%	96.90%
Scaffolded	671.726 Mbp	790	2.62 Mbp	212	403.346 Mb	39.06%	97.00%
Manual curation (reviewed)	671.695 Mbp	799	2.62 Mbp	219	245.409 Mb	39.06%	97.00%
Gap-closed	671.687 Mbp	772	2.8 Mbp	219	245.411 Mb	39.06%	97.00%
Repeat-masked (final)	671.687 Mbp	772	2.8 Mbp	219	245.411 Mb	39.06%	97.00%

Our *Ae. togoi* genome assembly is notably smaller than other *Aedes* mosquito genome assemblies (Figure 2D). We evaluated the completeness of our genome assembly using two complementary approaches: a k-mer analysis of raw sequencing reads and a scan for BUSCO single-copy orthologues. Both approaches suggest that our assembly is highly complete, with an estimated genome size of 589.75 Mb (Figure S1) and an overall BUSCO score of 97.0% (96.3% complete and single-copy, 0.7% complete and duplicated, and 0.9% present but fragmented; Table 1).

### Synteny with other mosquito genomes

The order of orthologous BUSCO genes in *Ae. togoi* was used to infer synteny with other *Aedes* species with chromosome-scale genome assemblies. Much of the *Ae. togoi* genome is composed of large blocks that are syntenic to the *Ae. notoscriptus*, *Ae.* *aegypti*, and *Ae. albopictus* genomes, either in the same orientation (shown in blue) or inverted (shown in red) (Figure 2D). The *Ae. togoi* genome appears to match closely with the chromosome structure of *Ae. notoscriptus* (Figure 2D). The exception is at chromosome 2, where the arms around the centromere are in opposite positions when comparing *Ae. togoi* to the proposed *Ae. notoscriptus* assembly (Figure 2D). As compared to *Ae.* *aegypti*, there is a chromosomal translocation across chromosomes 2–3 (Figure 2D).

### Repeat sequence and protein-coding gene set prediction

Repeat content was estimated to represent 63.71% of the *Ae. togoi* genome, with 17.67% as DNA transposons, 13.88% as retroelements (LTRs and LINEs), and 30.78% as unclassified repeat regions (Figure 3A; Table S3A). To inform the prediction of protein-coding gene models, we used poly-A selected RNA-seq data from adult heads, adult bodies, whole larvae, dissected larval anal papillae, and dissected larval salt gland. The BRAKER3 automated annotation tool predicted 18,391 protein-coding transcripts encoded by 12,977 genes. Functional annotation of the *Ae. togoi* gene set was performed by assigning GO terms, PFAM domains, and inferring orthologs based on protein sequence similarity to *Ae.* *aegypti* and *Ae. camptorhynchus*. This resulted in 16,819 transcripts with GO term and PFAM annotations and 17,773 transcripts with orthology annotations (Figure 3B; Table S4). Using *Ae.* *aegypti* proteins as a reference database for whole-transcriptome BLAST analyses, 11,845 transcripts matched through reciprocal best hit (RBH) and were identified as putative 1:1 orthologues, while an additional 5,610 transcripts matched through best hit (BH) as putative orthologs (Figure 3B). Using *Ae. albopictus* proteins as a reference database for whole-transcriptome BLAST analyses, 11,720 transcripts matched through reciprocal best hit (RBH) and were identified as putative 1:1 orthologues, while an additional 5,850 transcripts matched through best hit (BH) as putative orthologs (Figure 3B). Using *Ae. camptorhynchus* proteins as reference, 11,822 orthologues were identified through RBH BLAST, and an additional 5,624 putative orthologs were identified through BH BLAST (Figure 3B). 10,243 of these *Ae. togoi* protein annotations had matches identified through RBH to all three reference species, while 4,244 only had annotation matches identified through BH (Figure 3B).

**Figure 3:**
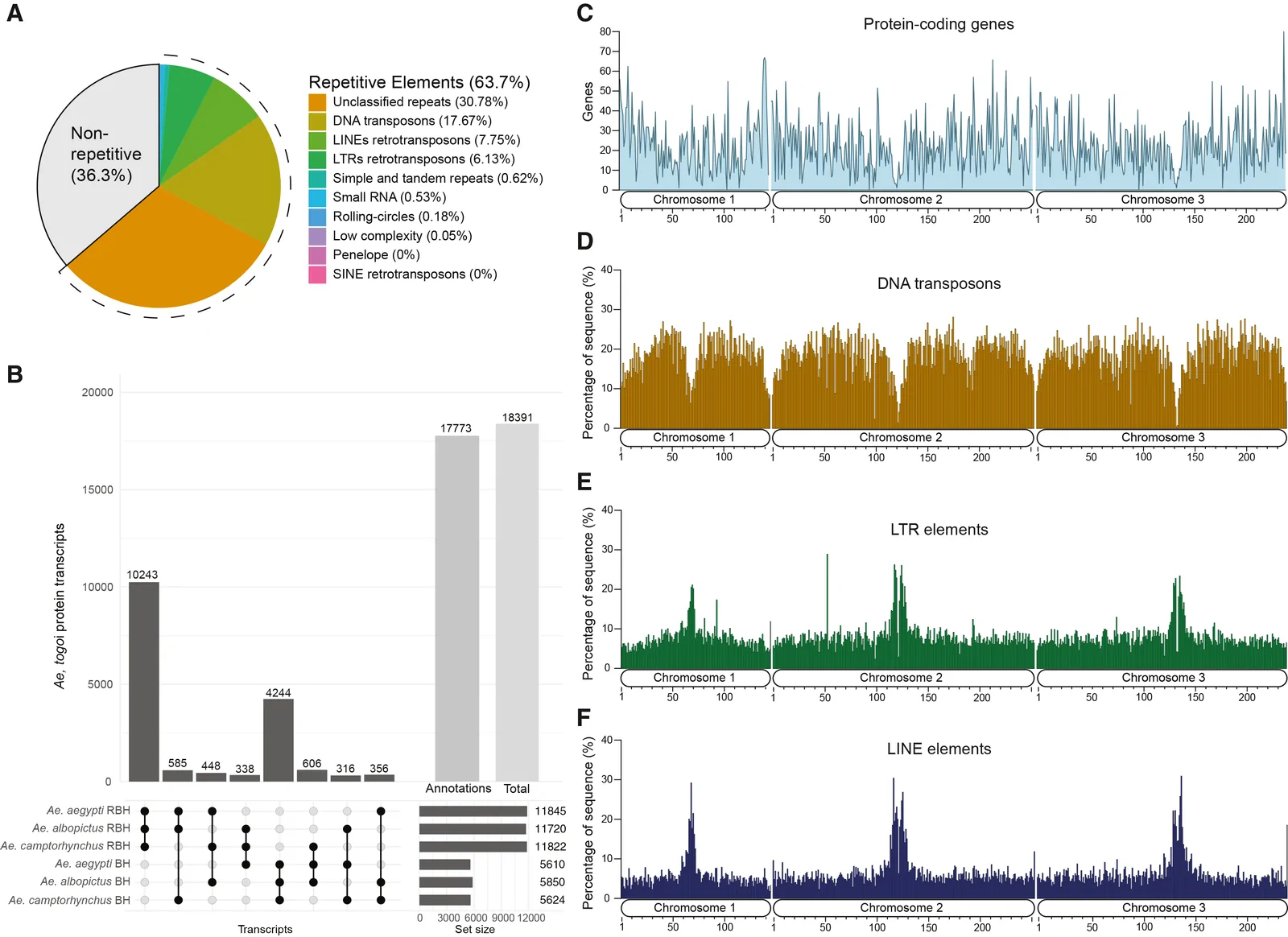
Annotation of protein-coding genes and repetitive elements in the *Aedes togoi* genome (A) Repetitive element composition of the Ae. togoi genome, estimated using RepeatModeler and RepeatMasker. The pie chart shows all repetitive content in the genome, separated by repetitive element classification. (B) Upset plot outlining the annotation of *Ae. togoi* proteins based on BLAST alignment to previous annotations in *Ae.aegypti*, *Ae. albopictus*, and *Ae. camptorhynchus*. Counts for predicted protein transcripts (Total), protein transcripts with an annotation (Annotated), protein transcripts matched through reciprocal best-hit (RBH) or best-hit (BH), and combinations of annotation match types are shown. (C–F) Proportion and distribution of (C) protein-coding genes, (D) DNA transposons, (E) long terminal repeat (LTR) retrotransposons, and (F) long interspersed nuclear element (LINE) retrotransposons along *Ae. togoi* chromosomes. Percentage of sequence in (C–F) was calculated by the proportion of sequence taken up by genes and repeat elements within 1 Mb stretches along the chromosomes.

### Distribution of genomic features across the three *Ae. togoi* chromosomes

Of these protein-coding genes, 12,917 (99.5%) were placed relatively evenly distributed on the three chromosome scaffolds, leaving 60 (0.5%) across the remaining genomic scaffolds (Figure 3C). BUSCO scores of the proteins encoded by these gene model predictions were similar to those measured at the genome scale, with 98.0% (3220 of the 3285) proteins identified as present and complete. Of these, 3195 had single copies, and 25 were duplicated, while the remaining BUSCOs included 5 fragmented and 60 missing. Repeat regions within each element type are relatively evenly distributed across the three chromosomes; however, in DNA transposons there is a noticeable decrease in abundance around peri-centromeric and telomeric regions (Figure 3D), while the abundance of LTR (Figure 3E) and LINE (Figure 3F) retrotransposons spike around peri-centromeric regions.

### GO term enrichment of transcripts in salt gland of the larval hindgut

Armed with these new genomic resources, we sought to investigate potential salt-secreting mechanisms in *Ae. togoi*. Unlike other saline-tolerant mosquitoes in the sub-genus *Ochlerotatus*, where the salt gland is a specialized segment situated just posterior to the typical rectum, the salt gland of *Ae. togoi* larvae is an elaboration of the anal canal that is not associated with the rectum (Figure 4A).^6^^,^^19^ To shed light on the potential ion-transport mechanisms for urine concentration utilized within the salt gland, we generated transcriptomes of this specialized organ from larvae reared in freshwater (FW) or seawater (SW). To identify transcripts enriched in the salt gland relative to the whole larvae, we also generated transcriptomes using tissues from FW- and SW-reared whole larvae. This is the first transcriptome of the specialized salt gland for a saline-tolerant mosquito larva.

**Figure 4:**
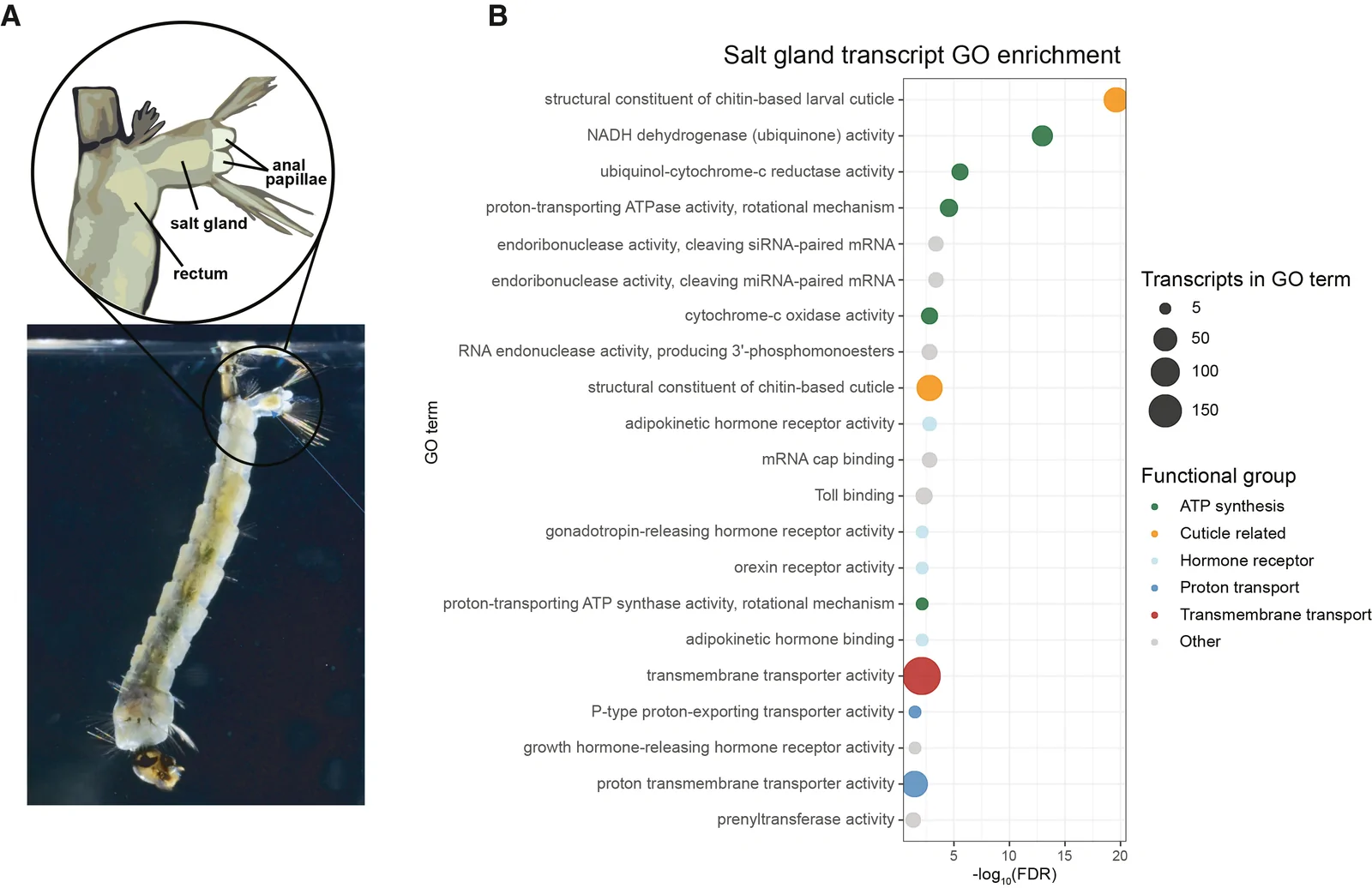
The salt gland of *Aedestogoi* acts as the primary site of salt ion excretion (A) The elaborated anal canal in *Ae. togoi*, the specialized salt-gland structure, in distinction from the rectum and anal papillae, ion regulatory structures. (B) Molecular function (MF) GO terms enriched amongst transcripts with high expression in the salt gland, compared to the transcripts expressed in the salt gland and whole larvae. Significance of enrichment is measured by false discovery rate (FDR), -log_10_ transformed in the plot. The enriched GO terms are grouped together by broad functional categories: ATP synthesis, cuticle related, hormone receptor, proton transport, transmembrane transport, and other.

To identify broad but specialized functions of the salt gland, we first used GO-term enrichment analysis targeting transcripts that are more highly expressed in the salt gland transcriptome as compared to the whole larval transcriptome (details in STAR Methods). We identified 2346 transcripts more highly expressed in the salt gland transcriptome (Table S5A). When compared to a background of 13995 transcripts expressed across the whole larval and salt gland transcriptomes (Table S5B), this yielded 21 significantly enriched GO terms (Figure 4B; Table S5C). GO-terms related to larval cuticle structural constituents (GO:0008010, GO:0005214), ATP synthesis (GO:0008137, GO:0008121, GO:0004129, GO:0046933, GO:0046961), proton transport (GO:0008553, GO:0015078), and transmembrane transport (GO:0022857) stood out as ones relevant for transmembrane ion transport (Figure 4B; Table S5C). Several GO terms related to hormone receptor activity were also identified (adipokinetic hormone receptor activity (GO:0097003), gonadotropin-releasing hormone receptor activity (GO:0004968), orexin receptor activity (GO:0016499), adipokinetic hormone binding (GO:0097004), and growth hormone-releasing hormone receptor activity (GO:0016520)) (Figure 4B; Table S5C). Together, these analyses provide insights into the types of transcripts enriched in this anatomically distinct organ.

### Impacts of high salinity on transcript expression in whole larvae and salt gland

Transcript expression from larvae reared in FW versus SW conditions was used to identify transcriptional responses to salinity stress in the whole animal and within the salt gland, the presumed site of hyperosmotic urine formation. There was noticeable differential expression in whole larvae reared in SW: 1470 transcripts were differentially expressed, 565 up-regulated in salt-water, and 905 down-regulated in salt-water (Figure S2A; Tables S6A–S6C). However, there were no significantly enriched GO terms among these differentially expressed transcripts in the SW condition compared to the FW condition at the whole larval level (Table S7). The two highest up-regulated transcripts (greatest log2FoldChange) were sodium/potassium transporting ATPase subunits (g7279.t1 and g7279.t9), both with low-moderate TPM expression in SW and zero TPM expression in FW (Table S6B). From a sub-list of 314 transcripts relevant to ion regulation and osmoregulation (see STAR Methods for description of how these were curated), 35 were differentially expressed in whole larvae reared in SW (Table S8; Figure S2B). These differentially expressed transcripts may be relevant for ion regulation in the epithelia of osmoregulatory organs but this cannot be confirmed using the whole larval tissue dataset, due to the lack of tissue-specificity. Therefore, we focused our attention primarily on the salt gland transcript expression dataset for insights on ion regulatory responses to salinity stress.

Through differential expression analysis of the *Ae. togoi* salt gland in FW and SW we have identified 886 transcripts that are differentially expressed: 426 up-regulated and 460 down-regulated in SW (Figure S2C; Tables S6D–S6F). We conducted GO-term enrichment of differentially expressed salt gland transcripts from larvae reared in FW and SW. Comparing SW and FW rearing conditions, we identified nine GO terms significantly enriched in the SW condition, five of which are related to transmembrane transport activity (Table S9A). Further, we found that only one of these GO terms (voltage-gated potassium channel activity; GO:0005249) is enriched among up-regulated transcripts in SW conditions, compared to up-regulated transcripts in the FW condition in the salt gland (Table S9B). We also examined the sub-list of 314 transcripts relevant to ion regulation and osmoregulation for differential expression in the salt gland and found 21 in total: 9 up-regulated and 12 down-regulated (Table S8; Figure S2D).

### Generating a hypothetical model of salt excretion in the *Ae. togoi* salt gland

We further examined expression of these ion regulatory transcripts in the salt gland, in both FW and SW conditions, inclusive of ion pumps and enzymes, transporters, ion and water channels, and paracellular junctions (Figures 5A–5E; Table S8).

**Figure 5:**
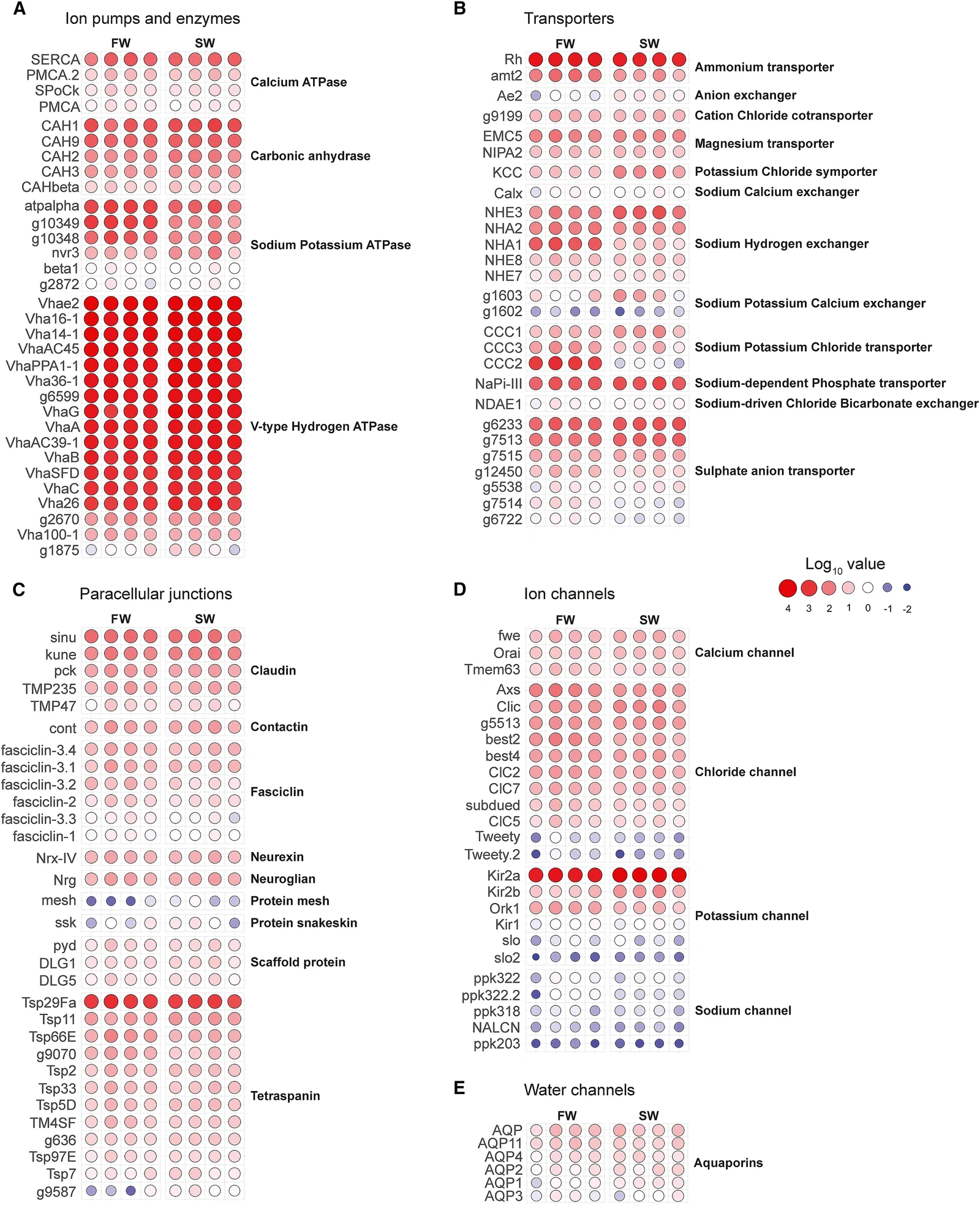
Abundance of ion regulatory-related transcripts in the salt gland of *Aedes togoi* larvae Ion-regulatory transcripts in the salt gland of freshwater (FW) and 100% seawater (SW) reared *Ae. togoi* larvae, organized by (A) ion pumps and enzymes, (B) transporters, (C) paracellular junctions, (D) ion channels, and (E) water channels. (A–E) Each group (FW and SW) features four replicates for all genes, showing transcript abundance represented by the log TPM value (log10 of TPM > 0.01) scaling with both size and color.

Among ion ATPases and other key enzymes, both calcium ATPases and carbonic anhydrases showed high expression with low differential expression across the FW and SW conditions (Figure 5A; Table S10A). Similarly, multiple subunits of the vacuolar-type hydrogen ATPase (VHA) were very highly expressed in both FW and SW conditions. On the other hand, several subunits of sodium-potassium ATPase (NKA) (atpalpha, g10329, g10348, and nvr3) showed high expression in FW conditions and decreased expression in SW conditions, while others showed consistently low expression (beta1 and g2872) (Figure 5A; Table S10A).

Many ion transporters show similar expression in the salt gland of larvae reared in FW and SW conditions, including the ammonium transporters, cation chloride cotransporters, magnesium transporters, sodium potassium calcium exchangers, and sodium chloride bicarbonate exchanger (Figure 5B; Table S10B). Interesting exceptions are the potassium-chloride symporter (KCC), sodium-hydrogen antiporters and exchangers (NHA and NHE), and sodium-potassium-chloride cotransporters (CCC) (Figure S2D). KCC is lowly expressed in FW but is up-regulated 2.5-fold when larvae are reared in SW. Among sodium-potassium-chloride-cotransporter paralogs, expression differs significantly; CCC1 is significantly up-regulated in SW while CCC2 and CCC3 are significantly down-regulated in SW compared to development in FW. CCC2, in particular, exhibits a significant decrease in transcript expression, from an average count of 830 TPM when in FW to 0.5 TPM when larvae are reared in SW (Figure 5B; Table S10B). Paralogs of the sodium-hydrogen exchanger (NHA/NHE) are differentially expressed in SW, notably with NHE3 up-regulated by 1.6-fold and NHA1 down-regulated by 5-fold in SW. Additionally, both the sulphate-anion transporters (g6233 and g7513) and a phosphate transporter (NaPi-III) are highly expressed in SW and are moderately up-regulated with a 0.7 and 0.5-fold increase, respectively (Figure 5B; Table S10B).

Cell-to-cell occluding junctions, or septate junctions, of invertebrate epithelia are a significant route for selective, transepithelial water and solute flux between cells. Septate junction proteins in the salt gland of larvae showed a wide range of expression but no differential expression between FW and SW conditions (Figure 5C; Table S10C). Certain protein transcripts were highly expressed, for example, Tsp29Fa in the tetraspanin family and the claudin proteins sinu and kune (Figure 5C; Table S10C).

Ion channel protein expression in the salt gland did not differ between FW and SW rearing conditions (Figure 5D; Table S10D). Sodium channels (ppk203, ppk322, ppk322.2, ppk318, and NALCN) had low expression, while calcium channels (fwe, Orai, Tmem63) were relatively moderately expressed overall (Figure 5D; Table S10D). Potassium channels exhibit a range of expression patterns, with Kir1, slo, and slo2 transcripts showing low expression and Kir2a, Kir2b, and Ork1 transcripts showing relatively high expression (Figure 5D; Table S10D). The Kir2a transcript, in particular, is highly expressed in both FW and SW. In addition, potassium (K^+^) inward rectifier (Kir) channels Kir2a and Kir2b are differentially expressed in the salt gland, with expression of these two transcripts increasing in SW by 1.7- and 1.5-fold, respectively. Specific chloride channels are also moderately expressed in the salt gland, except Tweety and Tweety-like, which have low transcript expression (Figure 5D; Table S10D). Water channels (i.e., aquaporins) were not differentially expressed in the salt gland between FW and SW conditions, and none of the identified aquaporin transcripts were highly enriched in the salt gland (Figure 5E; Table S10E).

### NHE3 isoforms sequence and transcript expression divergence

NHE3 is a sodium/proton exchanger expressed as two isoforms, one full-length protein and one with a shorter C-terminal tail,^36^ and has been described as a key component of larval sodium secretion in the euryhaline mosquito *Ae. taeniorhynchus*.^16^ There, it has been suggested that the shorter isoform may be crucial for higher sodium secretion rates required for *Ae. taeniorhynchus* larval survival in high salinity conditions,^16^ due to phosphorylation sites in the C-terminal tail having been implicated in regulating the number of NHE3 exchangers present on the cell membrane.^36^^,^^37^

Our orthology search identified two *Ae. togoi* transcripts matching the two described isoforms of NHE3 in other species: g10393.t2 (long NHE3 isoform) and g10393.t1 (short NHE3 isoform) (Figures 6A and 6B). Amino acid sequences of these isoforms were used to predict 12 transmembrane domains in the N-terminal half, and protein kinase A (PKA) phosphorylation-binding domains in the C-terminal half in both proteins (Figures 6A and 6B). Amino acid sequence alignment of the two isoforms revealed four deletion regions in the shorter NHE3 isoform protein, two on the N-terminal side and two on the C-terminal side (Figure 6B). The N-terminal side deletions do not remove any of the predicted transmembrane domains; however, the C-terminal side deletions result in four missing phosphorylation-binding domains in the shorter NHE3 isoform protein (Figure 6B).

**Figure 6:**
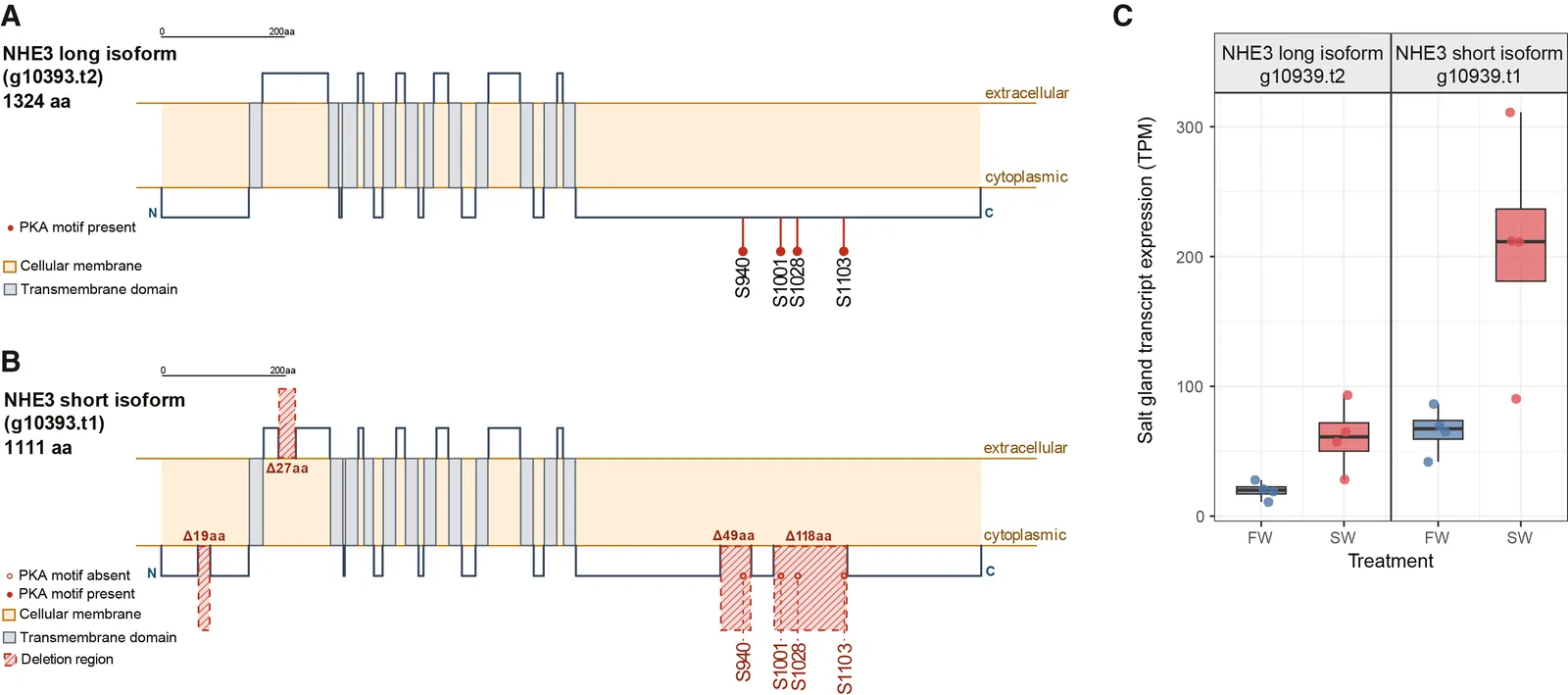
*Aedes togoi* NHE3 isoform sequence differences and transcript expression (A) Diagram of *Ae. togoi* NHE3 long isoform protein with predicted transmembrane domains and select phosphorylation binding domains shown. (A-B) Predicted transmembrane domains (gray) are shown in the expected position along a cellular membrane (beige). Only predicted phosphorylation-binding domains with protein kinase A (PKA; red) motifs identified in the *Ae. togoi* long isoform but not in the *Ae. togoi* short isoform are shown. Predicted phosphorylation binding domain names are given according to the amino acid placement of the phosphorylation site in the long isoform protein. (B) Diagram of *Ae. togoi* NHE3 short isoform protein with predicted transmembrane domains and phosphorylation binding domains missing due to deletions in the short isoform. NHE3 short isoform protein is shown plotted along *Ae. togoi* NHE3 long isoform coordinates to support visualization of amino acid sequence deletions. Amino acid sequence deletions found in the short isoform are shown in red-crossed regions. (C) Transcript expression of NHE3 long (g10393.t2) and short (g10939.t1) isoforms in salt glands of *Ae. togoi* larvae reared in freshwater (FW) or seawater (SW). Box represents the 25% and 75% range, with the median indicated by a horizontal line. Whiskers extend to the furthest point within 1.5× interquartile range.

NHE3 transcript expression differs between the two isoforms in the salt gland, with the short NHE3 isoform having higher TPM expression than the long isoform in both SW and FW conditions (Figure 6C). Although the short NHE3 isoform is expressed more highly (206.1 TPM mean across replicates) than the long NHE3 isoform (60.1 TPM mean across replicates) in the SW condition, both isoforms have a similar log2FoldChange up-regulation (∼1.65) in the SW condition compared to the FW condition.

## Discussion

Here, we present a *de novo* reference genome assembly for *Ae. togoi*, the first chromosome-scale assembly for a saline-tolerant mosquito. In addition, we provide transcriptomic insights into the mechanism by which this species tolerates high salinity and provide a putative model of salt secretion in the salt gland. These resources will support further studies to expand our understanding of mosquito genome evolution, develop genetic toolkits in lesser-studied branches of the *Aedes* genus, and characterize saline-tolerance strategies and mechanisms in primarily freshwater organisms.

### Chromosome re-arrangements within Aedes mosquitoes

Advances in genome sequencing technology, and heightened efforts to sequence a broader range of species, have transformed the way we can investigate mosquito genome organization. We used synteny to relate *Aedes* species and the ability of Hi-C data to identify points of mis-assembly to validate chromosome structures. Following scaffolding and manual curation, the chromosomal structure of our *Ae. togoi* genome assembly matches the overall structure of the *Ae. notoscriptus* (GCA_040801935.1) and *Ae. triseriatus* (personal communication with John Soghigian) genomes, based on BUSCO gene order showing syntenic arrangement of large blocks between these *Aedes* species (Figure 2D). The three largest scaffolds HiC_scaffold_101, HiC_scaffold_100, and HiC_scaffold_109, are observed to be chromosomes 1, 2, and 3, respectively (naming matched to *Ae. notoscriptus* chromosome numbers) (Figure 2D). There is one structural rearrangement in HiC_scaffold_109 that is not reflected in the matching *Ae. notoscriptus* chromosome 2 but it instead aligns with the arrangement of the *Ae. triseriatus* chromosome 2 (personal communication with John Soghigian) and is supported by our *Ae. togoi* Hi-C contact map signal patterning (Figure 2C; Figure S3). The concentration of alignment signal within scaffolds along the diagonal, other than the small regions of alignment signal between repetitive regions believed to be the chromosomal centromeres and telomeres (Figure 2C), and the lack of off-diagonal alignment signal, supports the scaffolding and manual rearrangement of chromosomes within our *Ae. togoi* genome assembly (Figure 2C; Figure S3).

Based on available data, chromosomal rearrangements are not uncommon within the *Aedes* genus and mosquitoes as a whole.^25^^,^^27^ For instance, comparing the previously assembled *Ae.* *aegypti* and *Ae. notoscriptus* genomes indicates large-scale rearrangement of chromosome arms (Figure 2D). Notably, syntenic blocks on the arms of chromosomes 2 and 3 are swapped with each other between these species, with half of *Ae.* *aegypti* chromosome 2 seemingly matching *Ae. notoscriptus* chromosome 2, and the other half matching to *Ae. notoscriptus* chromosome 3 (Figure 2D). However, we note that the publication describing the *Ae. notoscriptus* assembly does not present their Hi-C contact maps and has identified significant structural variation in chromosome 2 between populations, limiting our confidence in their proposed chromosome 2 structure.^38^ Additional high-quality, chromosome-scale genome assemblies from more species are required for a full picture of the evolution of chromosomal structure in the *Aedes* genus and across mosquitoes more broadly.

### Comprehensive gene-set annotations

A high BUSCO score of 98.0% for the predicted proteome sequences gives us confidence that the large majority of encoded *Ae. togoi* proteins are captured by our gene-set predictions. Further, with 96.6% (17,773 out of 18,391) of predicted protein transcripts matching previously annotated proteins in neighboring species (Figure 3B), we gain a baseline, sequence-level understanding of proteome function in this emerging study organism. Because few mosquitoes are saline-tolerant, and so few of these have high-quality genomic and transcriptomic resources, these annotations represent key points of reference in the landscape of saline-tolerance evolution in mosquitoes, potentially highlighting genomic similarities and protein characteristics common between saline-tolerant species. Therefore, these annotations provide a useful resource for future analyses characterizing specific genes and proteins and tracing the evolution of gene families, within the lens of understanding saline-tolerance lineages in particular.

### Relatively small genome size driven by reduced repeat content

At just 0.67 Gb, the *Ae. togoi* genome is among the smallest *Aedes* genomes assembled so far (Table S11). Compared to *Ae.* *aegypti*, which we estimated to have roughly 75.30% of its genome composed of repetitive content, the 63.71% repetitive content of the *Ae. togoi* genome represents a roughly 534.9 Mb (534,962,813 bp) reduction in genomic repeat sequence (Figure S4; Table S3A). Genome size is known to vary widely between species, and even within species between sub-populations, often due to differences in repetitive content.^39^^,^^40^ The relatively large genomes of *Aedes* mosquitoes are a prime example of this, as it is believed that the ballooning of repetitive elements has caused *Aedine* species such as *Ae.* *aegypti* and *Ae. albopictus* to have much larger genomes than species in other mosquito families such as *Anopheles* and *Culex*.^21^^,^^24^^,^^27^^,^^41^ This genome assembly for *Ae. togoi*, as a smaller *Aedes* genome with relatively low repetitive sequence content, adds an important reference point for understanding the dynamics of repetitive elements and their effects on fitness traits such as transcriptional gene regulation and phenotypic adaptability.

Transposable elements (TEs) in particular have been linked to genome size variation, genome rearrangements, and phenotypic adaptability to new environmental conditions, although their role in mosquitoes has only recently started to be outlined.^42^^,^^43^ Within Culicine mosquitoes, TE abundance is known to be high, with trends of retrotransposons being predominant over DNA transposons and there being an even distribution of TEs along chromosomes.^42^^,^^44^ Alternatively, we see that *Ae. togoi* DNA transposons (17.67% of genome) make up a greater proportion of genomic sequence than retrotransposons (13.88% of genome) (Figure 3A), in stark contrast to *Ae.* *Aegypti,* which (based on estimates with our described STAR Methods) has a strong leaning toward retrotransposons (44.57% of the genome) over DNA transposons (1.3% of the genome) (Figure S4; Table S3B). There is also a distinction in the distribution of transposable elements in *Ae. togoi*, in which retrotransposons (LINEs and LTRs) have greater abundance at peri-centromeric regions (Figures 3E and 3F) while DNA transposons have reduced abundance at peri-centromeric and telomeric regions (Figure 3D). This is distinct from previous results in *Ae.* *aegypti* and among Culicine mosquitoes, in which TEs are evenly distributed across chromosomes.^27^^,^^42^ However, one study reports increased abundance of LINE and LTR at peri-centromeric regions in *Ae.* *aegypti*, despite even distribution across all TEs.^45^ The patterns of LINE and LTR abundance observed in *Ae. togoi* are more characteristic of abundance patterns in *An. coluzzii* and *Cx. quinquefasciatus*, which both show high densities of LINEs and LTRs in peri-centromeric regions and low abundance elsewhere.^27^ In contrast, DNA transposons have decreased abundance at peri-centromeric and telomeric regions in *Ae. togoi*, which does not follow terminal inverted repeat (TIR) DNA transposon distribution patterns observed in *Ae.* *aegypti*, *An. coluzzii,* or *Cx. quinquefasciatus*^27^*.* These patterns of TE abundance and distribution may signify differences in the evolutionary history of *Ae. togoi* compared to other *Aedes* species studied so far and provide further insight into repeat element evolutionary history across Culicidae.

### Transposable elements and chromosomal inversions in adaptive trait evolution

Previous work assessing the influence of TE activity and chromosomal inversions in adaptive trait evolution points toward these as important factors in shaping genomes. Further work is required to understand how these factors have shaped the *Ae. togoi* genome but the differing composition of TEs (Figure S4) and the abundance of chromosome inversions in the genome (Figure 2D) may provide interesting insight into the evolution of this species.

TE activity has been previously implicated as a mechanism for rapid adaptation required to explain the genetic paradox of invasive species, in which species are able to rapidly adapt to new environments despite genetic bottlenecks.^43^^,^^46^ Further, it has been shown that stressful environmental conditions can trigger TE activity,^47^ and TEs have also previously been directly linked to the emergence of adaptive phenotypic traits such as insecticide resistance in *An. funestus* and cold-weather tolerance in *D. montana*.^48^^,^^49^ These together point to the potential that cold-weather and fluctuating water temperature and salinity in temperate, coastal regions may have played a similar role in triggering TE activity and adaptation in *Ae. togoi*.

Chromosomal inversions have also been identified as influential in the process of adaptive trait evolution and even speciation.^50^^,^^51^^,^^52^^,^^53^^,^^54^^,^^55^ In some instances, inversions can support adaptation to new environmental conditions by effectively reducing recombination rate within the inverted region and increasing the linkage between potentially beneficial alleles.^56^^,^^57^ In mosquitoes, inversions have been linked to specific adaptive phenotypic traits such as drought tolerance, insecticide resistance, anthropophilic feeding behavior, and changes to larval habitat.^54^^,^^58^^,^^59^^,^^60^^,^^61^^,^^62^^,^^63^^,^^64^^,^^65^ Interestingly, it seems that specific environmental conditions can foster distinct patterns of chromosomal inversions, resulting in the selection of similar traits affecting fitness in that environment.^66^^,^^67^ It is possible that chromosomal inversions have played an important role in the speciation and adaptation of *Ae. togoi* to tolerate cold weather and high salinity environments.

Further, there are potential interactions between TEs and chromosomal inversions, with evidence that TE activity promotes the occurrence of chromosomal inversions and possibly other types of chromosomal rearrangements.^68^ With the abundance of TEs and chromosomal inversions in mosquito genomes, it may be that these are primary and interactive drivers of adaptation in *Ae. togoi* and other mosquitoes.

Although this chromosome-level genome assembly provides an additional resource to study these interactions, more genome assemblies and further work comparing chromosome structure and repetitive element composition of many more mosquito species are required to fully understand these dynamics.

### Functional significance of enrichment in salt gland transcriptomes

GO terms enriched among transcripts more highly expressed in the salt gland highlight molecular functions important to the overarching role of this critical organ in salt excretion. Enriched GO terms were centered around larval cuticle composition, ATP synthesis, proton transport, and transmembrane transport, which together provide clues into how *Ae. togoi’s* extreme salt tolerance may be achieved (Figure 4B; Table S5C). It is well established that integumental adjustments of saline-tolerant insects include reduced permeability of the cuticle to attenuate water loss,^69^ and transcripts related to larval cuticle structure in our datasets highlight the importance of the cuticle as a barrier to avoid desiccation.^70^ Similarly, the enrichment of transmembrane transport transcripts was unsurprising and demonstrates the presence of ion transport machinery necessary to drive active salt secretion via the salt gland when larvae develop in seawater. Enrichment of proton transport GO terms is indeed exciting and suggests parallels with known mechanisms of hyperosmotic urine formation of *Ae. taeniorhynchus* larvae via H^+^-driven sodium secretion in the salt-secreting portion of the rectum.^16^ The high transcript expression of mitochondrial ATP-synthase subunit transcripts and V-type proton ATPase subunit transcripts (Table S8) together point toward proton gradients generated by proton ATPases as a mechanism for powering ion transport.

Additionally, several hormone-related GO terms are significantly enriched in the salt gland: adipokinetic hormone (AKH) receptor activity, gonadotropin-releasing hormone receptor activity, orexin receptor activity, adipokinetic hormone binding, and growth hormone-releasing hormone receptor activity (Table S5C). The enrichment of these GO terms comes almost entirely from enrichment of g10702 transcripts g10702.t1-g10702.t5 (Table S5C). Interestingly, these transcripts are annotated as AKH/corazonin-related peptide (ACP) receptor transcripts when referenced to *Ae. camptorhynchus* proteins but are annotated as gonadotropin-releasing hormone II receptor transcripts when referenced to *Ae.* *aegypti* proteins (Table S4). The ACP receptor is a structural intermediate between AKH and corazonin, which are two distinct insect neuropeptides.^71^^,^^72^ AKH is largely involved in energy homeostasis^73^ while corazonin has been shown to have a dual role in regulating metabolic and osmotic stress.^74^^,^^75^ ACP is localized to the brain of adult *Ae.* *aegypti* with axonal projections to the abdominal ganglia, which are enriched in ACP receptor transcripts.^71^^,^^76^ This is suggestive of a potential role of ACP in the regulation of release of other neuropeptides that are secreted from neurosecretory cells of the abdominal ganglia, specifically those with important roles in diuretic or anti-diuretic signaling pathways.^77^^,^^78^ The enrichment of a putative ACP receptor in salt gland tissue of larvae warrants a deeper investigation and might signify an important regulatory pathway when larvae are required to transition between antidiuresis and diuresis states as external salinity fluctuates.

Differential expression analysis of transcripts from the salt glands of larvae reared in FW and SW conditions, aiming to identify candidate ion transport processes critical for hyperosmotic urine production during high salinity, provided little information (Table S6). In fact, many transcripts that are implicated in active ion secretion in renal and extra-renal ion-transporting epithelia of insects are highly expressed in the salt gland of *Ae. togoi* larvae regardless of external salinity. Nonetheless, transcript expression abundance (TPM) revealed that many ion channels, transporters, and pumps are always highly expressed in this specialized organ. For example, V-type proton ATPase (VHA) subunits are very highly expressed in both FW and SW conditions (Table S8). This pump provides the driving force for transepithelial Na^+^ secretion from the hemolymph into the salt gland lumen of euryhaline *Ae. taeniorhynchus* larvae, and these data strongly suggest a parallel mechanism for Na^+^ secretion via the salt gland of *Ae. togoi*.^16^ Of course, transcript expression does not necessarily represent changes in VHA pump activity, and transcriptional regulation may not be the primary method for responding to changes in ion and osmoregulatory needs.^16^ In the renal Malpighian tubules of *Ae.* *aegypti* mosquito, VHA protein complexes are dissociated to achieve antidiuresis, and this phenomenon is under neuroendocrine control.^79^ As such, further studies that measure specific enzyme activities and that localize cytosolic and membrane protein complexes spanning dilute and hypersaline salinities are required. Post-translational methods of regulation, for example, through reversible dissociation or phosphorylation causing protein inactivation/reactivation^80^ may be productive avenues for future exploration.

### Transcriptional expression of the short NHE3 isoform

Our identification of two NHE3 isoforms, paired with our transcript expression data in FW and SW conditions, substantiate hypotheses that the short NHE3 protein might contribute to salt secretion in response to high salinity in euryhaline mosquitoes.^14^^,^^16^ Previous studies have suggested that the short NHE3 isoform may lack phosphorylation-binding domains, which, when removed, cause the short NHE3 protein to be regulated independently of the long NHE3 protein.^16^ Our analysis revealed four PKA phosphorylation-binding domains missing in the short NHE3 protein that may be key components in the regulation of long versus short NHE3 (Figure 6B). Phosphorylation by PKA has long been correlated with the regulation of NHE3 transport in vertebrates^81^^,^^82^^,^^83^ but it has not been examined whether this is also true in mosquitoes. If phosphorylation by PKA has a similar effect on NHE3 in mosquitoes, the lack of these PKA phosphorylation sites in the short NHE3 isoform could explain the elevated expression observed. Increased expression of the short NHE3 isoform, in SW compared to FW conditions, and relative to the long NHE3 isoform (Figure 6C), supports hypotheses presented in previous studies. However, further analyses verifying the connection between NHE3 transcript expression and sodium secretion activity, validating the inactivation effect of NHE3 phosphorylation by PKA, and examining the role of the four PKA phosphorylation sites missing in the short NHE3 isoform are needed to fully understand the role each NHE3 isoform has in euryhaline mosquito saline tolerance.

### Hypothetical cellular model of salt secretion across the salt gland epithelium from a transcriptomic lens

Numerous elegant and thorough studies over the past 80 years have implicated the salt gland of euryhaline mosquito larvae as the primary site of active salt secretion from the blood and into the urine to void excess salt as a strategy to hydrate.^6^^,^^14^^,^^16^^,^^84^ Therefore, it was exciting to find that the transcripts of many conserved solute and water carrier proteins critical for transepithelial membrane transport in insect osmoregulatory epithelia are highly enriched in our *Ae. togoi* salt gland transcriptome datasets. It seems that in lieu of transport novelty, saline tolerance by *Ae. togoi* larvae was probably achieved using machinery typical of ion-secreting epithelia such as the insect renal Malpighian tubules. Furthermore, we identify parallels in enriched membrane proteins with the salt gland of *Ae. taeniorhynchus* despite the differences in hindgut anatomy. Here, we develop a putative model for ion flux across the salt gland epithelium of *Ae. togoi* larvae that draws on this wealth of literature from fly epithelial ion transport. Although this is based on transcriptomic information, we hope that this working cellular model serves as a fruitful avenue for future hypothesis-driven testing and characterization of the molecular underpinnings of hyperosmotic urine production.

Here, we propose a working cellular model of transepithelial ion secretion across the salt gland epithelium of *Ae. togoi* (Figure 7), using the expression of ion-regulatory transcripts enriched in the salt gland of larvae in SW and leaning on the well-characterized transport models of ion flux from other mosquito osmo- and ion-regulatory organs including the Malpighian tubules, rectum, and anal papillae.^85^^,^^86^^,^^87^^,^^88^^,^^89^^,^^90^^,^^91^^,^^92^ Drawing from the mechanism for Na^+^ secretion described for the salt gland of *Ae. taeniorhynchus*,^16^ we propose that the vacuolar-type hydrogen pump (VHA) is expressed on the apical membrane and pumps protons toward the lumen, thereby contributing to a negative cytosolic voltage and establishing a H^+^ gradient as a driving force for the secretion of other ions (Figure 7). We acknowledge that this is transcript expression and that the localization of VHA within the epithelium remains to be determined; however, the degree to which it is enriched and the fact that VHA is almost always expressed on the lumen-facing apical membrane strongly suggests that this is the case for the salt gland of *Ae. togoi*. Given the high expression of ammonium transporters (amt2, Rh), apical expression of VHA likely facilitates an acid-trapping mechanism for nitrogenous waste (NH_3_/NH_4_^+^) excretion from the hemolymph, as is the case for many excretory organs of mosquito larvae, including the anal papillae of freshwater-restricted species *Ae.* *aegypti*.^86^ We propose the protons for VHA are supplied by cytosolic carbonic anhydrase (CA) (Figure 7), given the high expression of multiple CA isoforms and previous work in *Ae.* *aegypti* highlighting a similar role in the anal papillae.^86^^,^^87^^,^^89^

**Figure 7:**
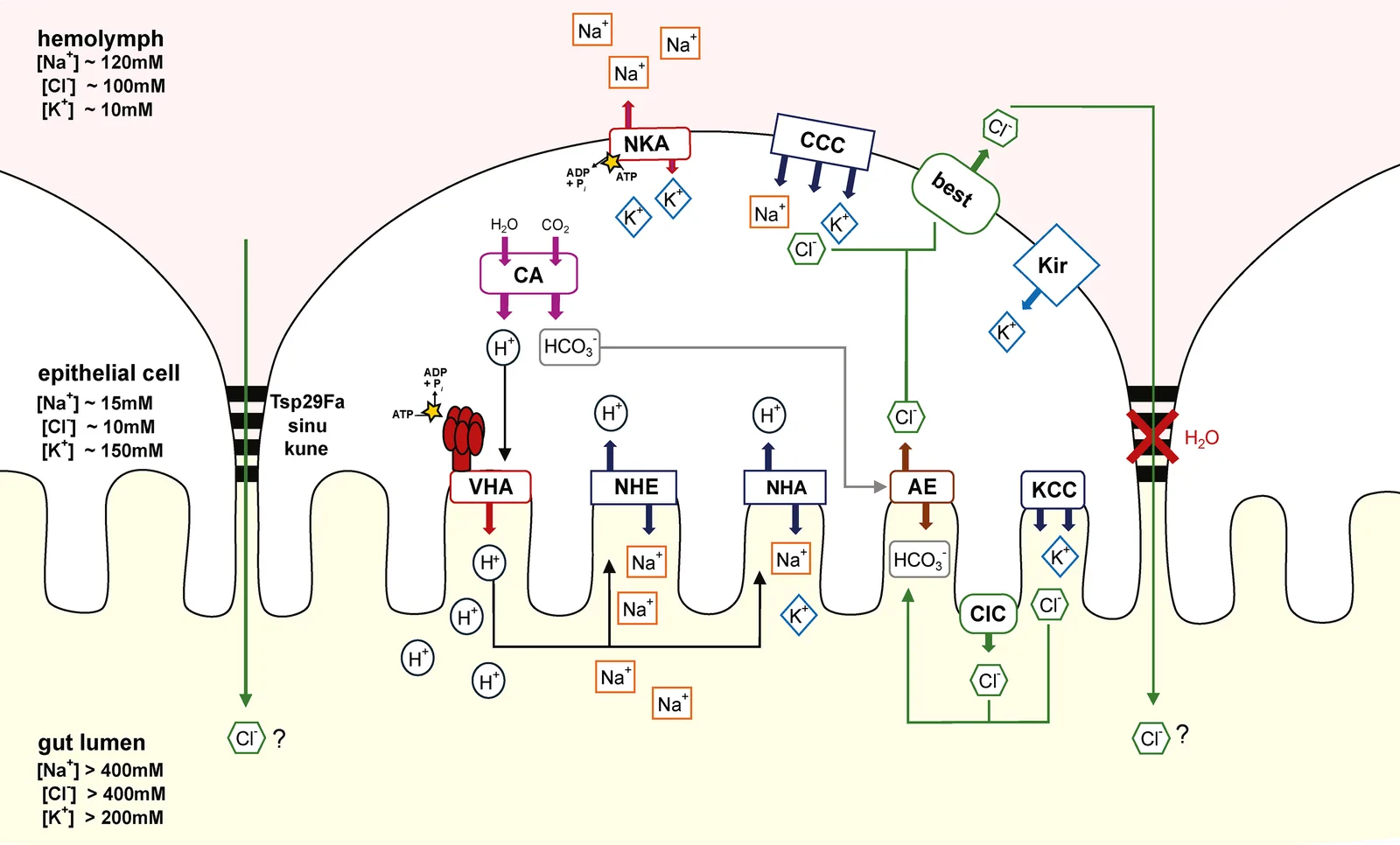
Putative model of sodium and chloride secretion in the salt gland of *Aedes togoi* larvae Working cellular model of sodium (Na^+^) and chloride (Cl^−^) secretion by the salt gland of the hindgut of *Ae. togoi* larvae. A putative model of salt secretion from the hemolymph, across the salt-gland epithelium, and into the lumen is presented using enriched sequences. On the apical membrane, the V-type H^+^-ATPase (VHA, multiple subunits) pumps protons (H^+^) generated by carbonic anhydrases (CA, multiple isoforms) into the luminal space. High luminal H^+^ drives the secretion of mostly Na^+^, and some K^+^, apically into the lumen via the sodium-hydrogen exchanger (NHE) and an inwardly rectifying potassium channel (Kir) on the basolateral membrane. The potassium chloride co-transporter (KCC) and chloride channel (ClC) are low-resistance routes for apical K^+^ and Cl^−^ flux into the lumen, and this is driven by the K^+^ and Cl^−^ electrochemical gradients. Cl^−^ flux back into the cell cytosol across the apical membrane might also drive bicarbonate (HCO3^-^) secretion into the salt gland lumen, transported by an anion exchanger (AE). We suggest that basolateral Cl^−^ flux back into the hemolymph from the cytosol occurs via a low resistance pathway that involves a chloride channel (best). We propose that a major route for Cl^−^ flux into the lumen might be a low-resistance paracellular pathway, similar to other insect secretory epithelia. The positive luminal space created by the excess sodium ions drives the paracellular movement of chloride through water-impermeable septate junction protein complexes (sinu, kune, and Tsp29Fa). The active secretion of high levels of Na^+^, K^+^, and Cl^−^ across the salt gland epithelium, one that has low aquaporin expression and a high resistance to water flux, allows for the generation of a hyperosmotic urine by larvae to achieve ion and water balance when in seawater.

While sodium-potassium-ATPase (NKA) transcripts (Atpalpha) are highly up-regulated with SW rearing in whole larvae (Figure 5), NKA expression and pump activity are critical for many biological processes, and its function is ubiquitous in nervous, muscle, and epithelial tissues. We include NKA in our model of salt secretion as transcript levels of multiple subunits are enriched but we acknowledge that the salt gland in our dataset is comprised of epithelial, muscle, and probably nervous tissue that would each have NKA transcript expression. Given the notable lack of NKA protein expression in the salt gland epithelium of *Ae. taeniorhynchus* larvae,^16^ we do not rule out the possibility that NKA protein is not a critical energizer of transepithelial ion flux in the salt gland of *Ae. togoi* larvae. Instead, we propose that protons drive the secretion of mostly sodium and some potassium through highly expressed apical sodium hydrogen exchangers, NHE and NHA (Figures 5 and 6), similarly to what has been characterized for the salt gland of *Ae. taeniorhynchus* larvae.^16^ NHA is expressed in the stellate cells of the Malpighian tubules, suggesting NHA-mediated transport of both Na^+^ and K^+^ into the tubule lumen,^85^^,^^93^ and we hypothesize that these enriched transcripts in the *Ae. togoi* salt gland might be serving similar functions.

In the salt gland of *Ae. taeniorhynchus,* it was suggested that a basally expressed cation-chloride cotransporter (CCC) transports ions into the cytosol from the hemolymph,^16^ similarly to the principal cells of the Malpighian tubules of *Ae.* *aegypti*.^94^ Given that the different CCC isoforms have distinct expression patterns in the salt gland of *Ae. togoi* (Figure 5), it is very likely that a basally expressed CCC (CCC1) is a low-resistance route for electroneutral ion flux into the cytosol from the hemolymph (Figure 7). CCC1 has been suggested to facilitate ion secretion, whereas CCC2 and CCC3 might have critical functions in absorptive epithelia.^95^ The very high expression of the inward rectifier potassium channels (Kir2a and Kir2b) (Figure 5) likely also mediates potassium flux into the cytosol from the hemolymph, for secretion across the apical membrane via NHA and a potassium chloride cotransporter (KCC) (Figure 7). Kir2b is highly expressed in the principal cells of the Malpighian tubules and is critical for transepithelial potassium secretion.^91^ We propose that the enriched KCC is apically expressed, similar to the renal tubules of *Ae.* *aegypti* for the generation of primary urine using potassium as a major osmolyte.^90^

In this putative model of salt secretion, apical KCC is a low-resistance route for the removal of intracellular potassium toward the lumen, and also a path for chloride flux for secretion in the urine. We posit that luminal Cl^−^ is a primary driving force for HCO_3_^−^ secretion via an apically expressed anion exchanger (AE) (Figure 7). Intracellular bicarbonate is generated by cytosolic carbonic anhydrase (CA) and is concentrated in the cytosol as a by-product of cytosolic CA activity and the secretion of H^+^ by VHA (Figure 7). Bicarbonate ions are also concentrated in seawater that the larvae drink, and therefore, would likely need to be secreted from the hemolymph into the urine. The AE provides a low-resistance route for HCO_3_^−^ secretion, and this occurs via electroneutral exchange with luminal Cl-that is transported apically via the KCC and an apical chloride channel (ClC). In the anal papillae of freshwater *Ae.* *aegypti*, an AE is apically expressed in the epithelium along with a basally expressed chloride channel for chloride absorption.^89^ Here, we suggest a similar mechanism for transcellular chloride flux from the lumen and back into the hemolymph via a basally expressed chloride channel (best). We propose that the main route of chloride secretion into the urine for excretion is paracellular via septate junctions and driven by an electrical gradient established by high luminal cation levels (Figure 7). Paracellular chloride flux has been demonstrated in the Malpighian tubules, albeit to drive fluid secretion.^85^ Given the low expression of water channels (Figure 5) and the increased desiccation risk in seawater, we suggested that there is a very high resistance of the salt gland epithelium to transcellular or paracellular water flux (Figure 7). We also note that the septate junctional complex proteins claudins, sinu, kune, and tetraspanin (Tsp29Fa) that are enriched in the salt gland (Figure 5) play a large role in the development, regulation, and maintenance of the septa, and so they are very likely important in regulating paracellular chloride secretion.^96^

### Limitations of the study

We would like to acknowledge the limitations of the current study. First, we performed our transcriptomic studies of salt response at two salt concentrations, unlike the fluctuating and unpredictable salinities that *Ae. togoi* are likely to experience in the wild. However, we note that mosquito hemolymph is maintained in the range of 10-15ppt, meaning that anything above that (including the two concentrations used in this study) would require the engagement of salt-secretion mechanisms and, thus, we would predict that the transcriptional and functional responses would be similar under a wide range of hyperosmotic conditions. Second, our primary readout of salt gland function was based on bulk transcriptional responses at fresh- and salt-water conditions. This will not reveal any mechanisms of regulation at the translational or post-translational level, nor does it detail the cellular and sub-cellular localization within the whole larvae and the salt gland of *Ae. togoi*. Thus, a full mechanistic description of *Ae. togoi* osmoregulation will await future studies deploying physiological, immunohistochemical, biochemical, pharmacological, and/or functional genetic techniques. Finally, due to a paucity of resources and experimental data, it remains unclear which aspects of osmoregulation in *Ae. togoi* are unique to this species, relative to other euryhaline mosquitoes. Future work describing the genomic and functional basis of osmoregulation will be required to determine the degree to which salt-tolerance and osmoregulatory mechanisms are shared across mosquitoes.

With the addition of the *Ae. togoi* genome assembly, accompanying protein-coding annotations, and transcriptomic datasets, we support future comparative genomics and physiological studies seeking to understand the genetic and molecular mechanisms of mosquito physiology. It has been known for almost a century that salt glands of saline-tolerant *Aedine* mosquito larvae are necessary for hydrating in seawater but mechanistic information underlying urine concentration remains scarce. The salt gland transcriptomes and putative model of salt secretion open new doors to a comprehensive understanding of salt secretion that is fundamentally different from other insects in desiccating environments. These resources and findings establish *Ae. togoi* as a study species well-suited for characterizing ion regulation and salinity tolerance in mosquitoes.

## Resource availability

### Lead contact

Requests for further information and resources should be directed to and will be fulfilled by the lead contact, Benjamin Matthews (ben.matthews@ubc.ca).

### Materials availability

This study did not generate new unique reagents beyond data.

### Data and code availability

• Assembly and sequencing reads are accessible at NCBI under BioProject accession PRJNA1370975. Assembly, annotations, and additional information are available at https://github.com/bnmtthws/2026_Aedes_togoi_genome
• This paper does not report original code
• Any additional information required to reanalyze the data reported in this paper is available from the lead contact upon request

## Acknowledgments

We would like to acknowledge members of the Matthews lab who provided feedback and support throughout the course of this project, particularly Leisl Brewster and Elva Vidya who provided helpful comments on earlier versions of the text and figures. We would like to thank Nicholas K. Tochor for visualizations in figures presenting salt gland structure and the overview of genomic and transcriptomic sequencing and analysis. We would like to acknowledge technical assistance and guidance from Marco Todesco during the generation and interpretation of Hi-C data. Finally, we would like to thank John Soghigian for his open discussions of unpublished work and for his suggestion to curate *Ae. togoi* chromosome synteny based on *Ae. notoscriptus* and *Ae. triseriatus* chromosome structures. This research was supported in part through computational resources and services provided by Advanced Research Computing at the University of British Columbia.

This work was supported by a Postgraduate Scholarship-Doctoral (PGS-D) from the 10.13039/501100000038Natural Sciences and Engineering Research Council of Canada (NSERC) to EK; NSERC Discovery Grant RGPIN-2020-05423 to BJM; Michael Smith Health Research BC Scholar Award SCH-2021-1860 to BJM; Alfred P. Sloan Foundation Fellowship FG-2021-16383 to BJM; and Canada Foundation for Innovation and BC Knowledge Development Foundation Awards 40036, 41223, and 43348 to BJM.

## Author contributions

**J.C.** contributed to: conceptualization (genomic sequencing, genome assembly, genome annotation, transcriptomic analysis), data curation (processing, cleaning, and annotating of genomic and transcriptomic sequencing data), formal analysis (genome synteny, repeat content, gene annotation, protein orthology, transcript differential expression), investigation (genomic sequence data collection, genome assembly, genome annotation, genome content analysis, genome synteny analysis, repeat content analysis, transcriptomic analysis), methodology (genomic sequencing, genome assembly, genome annotation, genome content analysis, genome synteny analysis, repeat content analysis transcriptomic analysis), project administration, software, validation, visualization, and writing (original draft and review/editing). **E.K.** contributed to: investigation (performed experiments and data collection related to the salt-gland transcriptomics), methodology (salt gland transcriptomic analysis and salt-secretion model), data curation (processing, cleaning, and annotating of salt gland RNA-seq data), visualization (preparation and creation of figures for salt gland RNA-seq data and salt-secretion model), and writing (original draft for the salt gland transcriptomic analysis and salt-secretion model). **O.P.** contributed to: investigation, methodology, and formal analysis of rock pool salinity and temperature findings. **A.K.P.N.** contributed to: investigation (whole larvae RNA-seq data collection). **D.A.H.P.** contributed to: methodology and supervision of rock pool salinity and temperature findings. **A.C.D.** contributed to: conceptualization (salt gland transcriptomics), funding acquisition (salt gland RNA-seq), methodology (salt-secretion model), and writing (review/editing). **B.J.M.** contributed to: conceptualization (genomic sequencing, genome assembly, genome annotation, transcriptomic analysis), funding acquisition, project administration, resources (mosquito sample collection, instrumentation, genomic sequencing, computing resources), supervision, visualization (created species distribution and rock pool salinity and temperature figures), and writing (review/editing).

## Declaration of interests

The authors declare no competing interests.

## STAR★Methods

### Key resources table

**Table:** 

REAGENT or RESOURCE	SOURCE	IDENTIFIER
**Criticalcommercialassays**		

Monarch® HMW DNA Extraction Kit for Tissue	New England Biolabs	T3060
Arima High Coverage HiC Kit for Animal Tissues	Arima Genomics	A101030
RNeasy Mini Kit	Qiagen	74104
Direct-zol RNA MicroPrep	Zymo Research	R2060

**Depositeddata**		

Sequencing reads, Assembly	NCBI	BioProject PRJNA1370975
Annotation	GitHub	https://github.com/bnmtthws/2026_Aedes_togoi_genome

**Experimentalmodels:Organisms/strains**		

*Aedes togoi* laboratory colony	Lighthouse Park, West Vancouver, British Columbia, Canada	*Aedes togoi* LHP strain

### Experimental model and study participant details

#### *Ae. togoi* field work

All field work for this project took place in Lighthouse Park in the city of West Vancouver (49.3316° N, 123.2636° W). To investigate the factors that affect larval survival a variety of abiotic and biotic factors were investigated over the course of a year (April 2021-April 2022). Sixteen rock pools in the southwest region of the park were chosen as sample sites. These rockpools represented various potential breeding habitats for *Ae. togoi* as they varied in size, volume, salinity, temperature, and distance from the sea. All rock pools were visited once a week for one year and the salinity, pH, temperature, approximate volume, and larval abundance was measured during each visit.

The temperature and salinity of the individual rock pools were measured using a Fisherbrand salinity meter pen (catalog #15078202). pH was measured using a Fisherbrand pH pen (catalog #S35927). The technique for measuring volume of the pools was adapted from a previously published protocol.^97^ The total area of each rock pool was divided into smaller shapes (i.e., square, rectangle, triangle) and the area of these shapes was measured using a Mileseey brand laser measuring device. Two to three depth measurements were taken for each shape using a 2m fold up ruler and an average depth was calculated. The volume of each shape was calculated, and these volumes were summed to get an approximate volume for the rock pool. Because the volume of the rock pools was extremely variable, volume was recalculated each week. Depths were taken from the same point each week to ensure that how the volume was changing week to week was being accurately measured. Historic weather data such as temperature and precipitation were obtained from Environment and Climate Change Canada Meteorological Service of Canada (ECCC-MSC) at the Vancouver Harbor CS weather station (CWHC). The total precipitation in the previous week, previous two weeks, and previous three weeks were calculated and used as variables. Similarly, the temperature from the previous week, previous two weeks, and previous three weeks were summed to create historic temperature variables. Finally, a mosquito dipper (volume 300 mL) was used to measure relative larval and pupal abundance. The pool was visually inspected to see where the largest density of larvae and pupae was, and this is where the measurement was taken. A total of three dips were taken; larvae and pupae were replaced after each dip and the average number of larvae and pupae per dip was calculated.

#### Establishment and rearing of an *Ae. togoi* laboratory colony

All *Ae. togoi* mosquitoes used in this study were retrieved from our lab colony, originally established from >400 larvae and pupae collected from supra-littoral rock pools in Lighthouse Park in West Vancouver in the summer of 2021 (Figure 1A) with permission granted from the District of West Vancouver. *Ae. togoi* were initially reared in similar conditions to *Ae.* *aegypti* (14 h light, 10 h dark at temperatures of ∼27°C and RH of ∼80%). In 2023, our colony was gradually shifted to being reared in a standing incubator with a 16 h light, 8 h dark cycle, with daytime temperatures of 25° gradually falling to 21° during the dark period. Humidity was maintained around 60% with pans of standing water in the bottom of the incubator. Adults are given *ad libitum* access to 10% sucrose and fed defibrinated sheep’s blood (Hemostat Laboratories via Cedarlane, DSB500) using Hemotek feeders. Following blood-feeding, eggs were collected on seed germination paper lining a plastic cup containing dechlorinated tap water, and allowed to gradually dehydrate for 7 days, at which time eggs were hatched (see below) or stored for later hatching. Hatching was performed by submerging egg papers in dechlorinated tap water and were transferred post-hatch to 6–8 ppt seawater (Instant Ocean) for routine rearing of larval and pupal stages. Adults were allowed to emerge in 30 × 30 × 30 cm mesh cages (Bugdorm 4E3030) and were reared as mixed sex populations.

### Method details

#### High molecular weight (HMW) genomic DNA extraction, library preparation, and sequencing

To generate HiFi reads, HMW DNA was extracted from a single *Ae. togoi* pupa in October 2021 (generation 3 of laboratory colonization) using a modified version of the NEB Protocol for High Molecular Weight (HMW) DNA Extraction from Tissue, utilizing changes to reagent volumes outlined in the ‘Using Very Low Tissue Amounts’ section of the kit (T3060) product manual. The pupa was snap-frozen in dry ice and then gently ground using only up and down strokes, using a micro-pestle. 50 μL of NEB Tissue Lysis Buffer was added while grinding continued to break up any large pieces. Another 50 μL of NEB Tissue Lysis buffer was added to rinse tissue on the pestle into the tube. 10 μL of NEB proteinase K (800U/mL) and 4 μL of 10% SDS solution were added before incubating the sample at 56°C for 1 h. The thermomixer used for incubation was set to 500rpm agitation for the first 15 min, while the remaining 45 min had no agitation. 5 μL of NEB RNase A solution (20 mg/mL) was added before incubating the sample again at 56°C for 10 min with 300rpm agitation. 55 μL of NEB Protein Separation Solution was added before mixing the sample by slow repeated inversion of the tube for 1 min. The sample was centrifuged at 16,000 × g for 30 min, at 20°C. The clear, upper fraction of the sample containing DNA was transferred to another tube using a wide-bore pipette tip. Two NEB DNA Capture Beads and 90 μL of isopropanol were added to the sample before mixing on a vertical rotating mixer at 10rpm for 5 min. Our protocol then continued following the steps listed in the Monarch High Molecular Weight DNA Extraction Kit for Tissue manual, starting from Step 3 of ‘Part 2 HMW gDNA Binding and Elution’ section. Library preparation using the ultra-low DNA input workflow and PacBio HiFi sequencing on two SMRT PacBio Sequel II flow-cells were carried out by the University of Oregon’s Genomics and Cell Characterization Core Facility (GC3F). Reads from this dataset can be found at NCBI SRA (Table S2).

Nanopore reads were generated from four libraries, prepared from four *Ae. togoi* samples in October 2024 (approximate lab generation 20): a single female pupa, a single male pupa, a pool of 8 female pupae, and a pool of 8 male pupae. HMW DNA were extracted from each sample using the same protocol as outlined above for HMW DNA extraction for HiFi sequencing, except reagent volumes used differed for the pooled samples, on account of additional tissue in the sample. The reagent volumes used for the two pooled samples were: 300 μL of NEB Tissue Lysis Buffer total (150 μL while grinding, 150 μL for rinsing), 5 μL of 10% SDS solution, 10 μL of NEB Proteinase K solution, 5 μL of NEB RNase A solution, 150 μL of NEB Protein Separation Solution, 275 μL of isopropanol, and 62 μL of elution buffer (10 mM TRIS-HCL, 0.1 mM EDTA; pH 8.0). ONT library preparation and sequencing was carried out by the BC Genome Science Center, following standard Nanopore PCR-free multiplexed genome library construction without shearing, using one PromethION flow cell. Reads from this dataset can be found at NCBI SRA (Table S2).

#### Hi-C genomic DNA extraction and library preparation

Hi-C data was collected from a single male *Ae. togoi* pupa, following a modified protocol adapted from the Arima Genomics Arima High Coverage HiC Kit for Animal Tissues (Material Part: A410110; Document Part: A160162 v01) and Arima Library Preparation Module for Arima High Coverage Kit (Material Part: A101030, A303011; Document Part: A160186 v02) manuals. The sample was snap-frozen using liquid nitrogen, pulverized into a fine powder using a micro-pestle, and then mixed with 1 mL of 1× PBS solution. Our protocol continued following the steps listed in the Arima High Coverage HiC Kit manual starting from step 2 of ‘Crosslinking – Low Input’, continuing through to the HiC, quality control, and library preparation sections as stated. Cross-linked DNA was fragmented to the target 550-600bp range using an ME220 Covaris Focused-ultrasonicator with the following parameters: 40 W power, 10% duty factor, 1000 cycles per burst, 20 °C water temperature, 75 s duration. The Hi-C library was sequenced using one P2 flow cell on the Illumina NextSeq2000, aiming for 150x-depth, paired-end, 150bp length reads. Reads from this dataset can be found at NCBI SRA (Table S2).

#### RNA extraction, library preparation, and sequencing: Adults

mRNA from adult *Ae. togoi* males and females were profiled by separately collecting heads and bodies from four males and five females and preparing for RNA extraction by adding 450 μL of TRIzol LS and 150 μL of PBS solution to each sample of pooled mosquito heads or bodies. Samples were disrupted using an Omni Bead Ruptor Elite 19-040E at 6.0 m/s for 30 s and then incubated at room temperature for 5–10 min 130 μL of chloroform was added before homogenizing by vortex at max speed for 20–30 s and allowing to incubate at room temperature for 10 min for phase separation to occur. Samples were centrifuged at 15,000 *x g* for 15 min at 4°C. The aqueous phase of each sample was transferred to new tubes and then ∼250 μL of isopropanol was added. Samples were left to incubate at −20°C overnight. The next day, samples were centrifuged at 10,000 *x g* for 10 min at 4°C. Supernatant was removed from the tube, leaving the pellet behind. 1 mL of ice-cold ethanol was used to wash the pellet. Samples were mixed by vortex briefly. Samples were then centrifuged at 7,500 × g for 5 min at 4°C. Ethanol was removed from the tubes and then the tubes were left to air-dry for 3–5 min at room temperature. The RNA pellet was dissolved by adding nuclease-free water and then letting the samples sit on ice for 1 h. All samples were the mixed by vortex briefly before storing at −80°C. RNA samples were sent to Novogene for library preparation and sequencing. Each sample went through poly(A) enrichment and standard library preparation protocols. The libraries were sequenced on the Illumina NovaSeq X Plus series for paired-end, 150bp length reads. Reads from this dataset can be found at NCBI SRA (Table S2).

#### RNA extraction, library preparation, and sequencing: Whole larvae

mRNA was collected from whole *Ae. togoi* larvae reared in freshwater and saltwater conditions. Briefly, larvae were hatched and then reared in either dechlorinated Vancouver tap water or dechlorinated Vancouver tap water supplemented with increasing concentrations of artificially formulated seawater (Instant-Ocean; increasing by the equivalent of ∼100% sea-water per day, final concentration: ∼300% seawater), until they reached 4^th^ instar. Larval samples were dried and then placed in 350 μL of Qiagen RNeasy Lysis Buffer and 3.5 μL of 2-Mercaptoethanol, before using an Omni Bead Ruptor Elite to disrupt the larval tissue at 4.5 m/s for 30 s. Samples were further disrupted by passing tissue through a Qiagen QIAshredder Mini spin column before RNA extraction was performed using the Qiagen RNeasy Mini Kit according to the manufacturer’s protocol. RNA was then sent to GENEWIZ for library preparation and 150bp paired-end Illumina sequencing on the Illumina HiSeq 4000. Reads from this dataset can be found at NCBI SRA (Table S2).

#### RNA extraction, library preparation, and sequencing: Dissected larval salt glands

mRNA was collected from dissected salt glands of *Ae. togoi* larvae in August 2025 from the same colony of *Ae. togoi* transferred to the University of Washington (approximate generation from colonization 25). From hatching until RNA-extraction at the 4^th^ instar stage, four biological replicates (containing 55–65 salt glands from individual larvae each) per condition were reared in either dechlorinated Seattle tap water or dechlorinated Seattle tap water supplemented with 35 g/L sea salts (Instant-Ocean). Larvae reared in freshwater were dissected in *Ae.* *aeygpti* physiological saline solution, adjusted to pH 7.2, containing (in mmol L^−1^): 60 NaCl, 3 KCl, 5 NaHCO_3_, 0.6 MgSO_4_, 5 CaCl_2_, 25 HEPES, 10 Glucose, 5 Succinic acid, 5 Malic acid, 10 Citric acid tri-sodium salt, 3.37 L-arginine, 14.4 L-leucine, 8.74 L-histidine, 9.1 L-glutamine, and 5 L-proline.^98^ Larvae reared in seawater (SW) were dissected in a modified *Ae.* *aegypti* physiological saline adjusted to pH 7.8, containing (in mmol L^−1^): 110 NaCl, 10 KCl, 5 NaHCO_3_, 0.6 MgSO_4_, 5 CaCl_2_, 25 HEPES, 10 Glucose, 5 Succinic acid, 5 Malic acid, 10 Citric acid tri-sodium salt, 3.37 L-arginine, 14.4 L-leucine, 8.74 L-histidine, 9.1 L-glutamine, and 5 L-proline adjusted to a pH of 7.8, matching levels measured for *Ae. taeniorhynchus* in seawater. Salt gland tissue was sonicated for 5 s at amplitude 10 using a sonic dismembrator (Model 50, Fisherbrand) and a 1.8-inch probe (Model CL-18, QSonica). RNA was isolated using the Direct-zol RNA MicroPrep kit (Cat #: R2060, Zymo Research) and then sent to the Northwest Genomics Center (NWGC) for quality control (Quant-iT RNA assay from Invitrogen and fragment analyzer from Advanced Analytical), library preparation (Catalog: 20020595, TruSeq Stranded mRNA kit, Illumina), and sequencing (Illumina HiSeq 4000, 100bp paired end). Reads from this dataset can be found at NCBI SRA (Table S2).

#### Read QC and processing

HiFi reads were trimmed to remove adapter content using Lima (v2.13.0) and PCR-deduplicated with pbmarkdup (v1.2.0). HiFi, ONT, and Hi-C read quality were checked using FastQC (v0.12.1).^99^ Hi-C reads were trimmed to remove the front five base pairs using fastp.^100^ Genomescope2 (v2.0) was used to estimate heterozygosity, repeat content, and error rate of HiFi reads based on Meryl (v1.4.1) k-mer analysis (Figure S1).^101^^,^^102^

#### Genome assembly

The primary genome assembly was performed using Hifiasm (v0.24.0).^103^ HiFi reads were used as the primary input reads while additional integration of ultra-long ONT and Hi-C reads were used to improve contiguity and haplotype resolution, respectively. The resulting assembly of primary contigs was used for further assembly steps. Purging of haplotypic duplication was done using purge_dups (v1.2.5).^104^ The Arima Genomics mapping pipeline (A160156_v03) was used to deduplicate and align Hi-C reads to the purged contig assembly. Scaffolding was performed using YaHS (v1.2.2) with default parameters.^105^

The complete mitochondrial genome sequence was assembled using MitoHifi (v3.2.2), using default parameters with The Invertebrate Mitochondrial Code (-o 5).^106^ Mitochondrial genome features were annotated using Mitohifi and MITOS2 (v2.1,10).^107^

#### Gene-order synteny between aedes species

Syntenic blocks on analogous chromosome scaffolds between species were identified and visualized with chromsyn (v1.3.2) based on gene-order synteny of the locations of BUSCO single-copy orthologues.^108^ The *Ae. albopictus* (GCA_035046485.1), *Ae.* *aegypti* (GCA_002204515.1), and *Ae. notoscriptus* (GCA_040801935.1)^38^ genomes were accessed from the NCBI genome database.

#### Divergence time between aedes species

The four Aedes species used in gene-order synteny comparison were displayed on a chronogram with divergence times generated by pruning alltribes_posterior_20220716.trees, taken from Soghigian et al. 2023’s culicitree repository.^109^ All 100 trees were pruned to include just *Aedes* *aegypti*, *Aedes albopictus*, *Aedes notoscriptus*, and *Aedes togoi*. Divergence times presented are median values taken across all 100 pruned trees, with 95% credible intervals shown in square brackets.

#### Genome assembly curation and finalizing

Several changes to the YaHS scaffolded assembly were made by manual curation using the Juicer, Juicebox, and 3D-DNA suite of tools. Juicer (v2.0)^110^ was used to generate the Hi-C contact map based on the scaffolded genome assembly, which was visualized and manually edited using Juicebox (v3.1.4).^111^

Manual curation was conducted with guidance from gene-order synteny, alignment of ONT long-reads to the scaffolded *Ae. togoi* genome assembly, and Hi-C contact map signal patterns. Gene order synteny between *Ae. togoi*, *Ae. notoscriptus*, and *Ae. triseriatus* genomes (personal communication with John Soghigian), made it possible to identify instances of chromosome structure similarity and potential translocations and inversions (Figure S5). *Ae. notoscriptus* and *Ae. triseriatus* were chosen to guide curation of *Ae. togoi* chromosome scaffolds because these two species are more closely related to *Ae. togoi* than other *Aedes* species with chromosome-scale genome assemblies, like *Ae.* *aegypti* and *Ae. albopictus*.^112^ Syntenic blocks on *Ae. togoi* chromosome scaffolds that aligned with non-equivalent *Ae. notoscriptus* and *Ae. triseriatus* chromosome scaffolds were rearranged to agree with the *Ae. notoscriptus* and *Ae. triseriatus* chromosome structure, by breaking *Ae. togoi* chromosome scaffolds and rearranging chromosome fragments where needed in Juicebox (Figure S3). Inversions of regions within larger syntenic blocks were not edited. When a potential adjustment was identified, the sequence range for a potential scaffold cut-point was located by identifying the two BUSCO genes closest to either side of the point where the *Ae. togoi* scaffold starts to align with different scaffolds on the *Ae. notoscriptus* and *Ae. triseriatus* genomes (Figure S5). The region between the two BUSCO genes identifies the potential sequence range for the proposed cut-site, which is determined by analyzing Hi-C contact signal patterns, looking for irregularities that suggest mis-assembly at that region. A finer-resolution cut-site is identified within the potential sequence region by aligning ONT long-reads to the scaffolded assembly and looking for gaps in read coverage caused by poor read alignment, truncated reads, or N insertions in the genome assembly (Figure S6). In the situation of there being multiple potential cut-sites of equally low read coverage within the proposed region, the final decision was made based on the Hi-C contact signal pattern–specifically, potential cut-sites at regions with no or little signal were prioritized over potential cut-sites in regions of dense contact signal. Once an exact cut-site was identified, a 1 kb region encapsulating the cut-site was removed from the scaffold and moved to debris as a separate scaffold, thereby creating a break in the existing scaffold. The resulting chromosome fragments on either side of the cut were then rearranged to match the order of syntenic blocks in the *Ae. notoscriptus* and *Ae. triseriatus* genomes. Finally, the cuts and chromosome rearrangements were accepted if the resulting Hi-C contact signal pattern no longer had signs of mis-assembly (Figure S3). The overall chromosome structure of the *Ae. notoscriptus* and *Ae. triseriatus* genome assemblies agreed for chromosomes 1 and 3, but not chromosome 2 where the chromosome arms of *Ae. notoscriptus* appear syntenic to opposite chromosome arms of *Ae. triseriatus* (personal communication with John Soghigian). In this case, curation of the *Ae. togoi* chromosome 2 was guided solely by *Ae. triseriatus* chromosome 2 structure because that chromosome arrangement was well supported by our *Ae. togoi* Hi-C contact signal pattern (Figure S3). Attempts to guide *Ae. togoi* chromosome structure to match *Ae. notoscriptus* chromosome 2 arrangement result in Hi-C contact signal patterning that would suggest mis-assembly of our *Ae. togoi* chromosome 2. A total of five cuts were made: two in scaffold_1 to separate out chromosomes 1 and 3, another two in scaffold_1 to excise mitochondrial genome sequence, and one in scaffold_2 to reorder the arms of chromosome 2 (Figure S3). 3D-DNA’s post-review pipeline was used to apply changes on the edited assembly map to the scaffolded genome assembly and for re-scaffolding, following which the assembly’s scaffolds adopt the ‘HiC_scaffold_’ naming scheme.^113^

Finally, stretches of N sequences (gaps) in the scaffolded assembly were closed with TGS-GapCloser (v1.2.1), using HiFi reads as input for gap-filling and Racon as the error-correction module.^114^

The assembled mitochondrial genome sequence, excised from the main assembly, was identified as HiC_scaffold_219 following the re-scaffolding process. This mitochondrial genome scaffold was separated from the rest of the genome assembly.

#### Genome assembly statistics

Genome assembly stats were measured using BBMap ‘stats.sh’ (v35.85).^115^ Genome completeness was measured by running BUSCO (v5.5.0),^116^ for conserved gene-level estimation with dipteran databases as reference.

#### Repeat content annotation

Repeat sequence composition was estimated by using RepeatModeler (v2.0.6) to create a *de novo* predicted repeat library and RepeatMasker (v4.1.9) to search for and mask repeat sequences.^117^^,^^118^ The accuracy of this methodology was somewhat validated by estimating the repeat content of the *Ae.* *aegypti* genome in the same way and finding an estimated 75.30%, comparable to previous estimations of 65–78%^24^^,^^25^ (Figure S4; Table S3).

#### Protein-coding gene set annotation

BRAKER3 (v3.0.8)^119^ was used to identify protein-coding regions within the soft-masked *Ae. togoi* genome assembly, utilizing the OrthoDb v10 Arthropoda ortholog database as inputs^120^ and RNA-seq libraries from *Ae. togoi* adult male heads and bodies, adult female heads and bodies, whole larvae, and larval salt glands, as described above, as well as data from larval anal papillae (to be described elsewhere).

#### Functional annotation

Annotation of protein coding genes, and their transcript and protein products, was done based on protein sequence similarity to *Ae.* *aegypti*, *Ae. albopictus*, and *Ae. camptorhynchus* proteins, three other *Aedes* genus species with previously annotated reference genomes.^24^ *Ae.* *aegypti* (NCBI Aedes aegypti Annotation Release 101), *Ae. albopictus* (GCF_035046485.1-RS_2024_01) and *Ae. camptorhynchus* (GCF_037179485.1-RS_2024_05) protein sequences were taken from NCBI Genome Annotation databases. Additional *Ae.* *aegypti* annotations curated through Mosquito Cell Atlas were also included.^121^ First, a search for high-confidence one-to-one orthologues was conducted using Orthologr’s blast_rec function (v0.4.2) to search for reciprocal best hits (RBH) between the reference species’ proteome and the BRAKER3-predicted *Ae. togoi* protein sequences.^122^ Additionally, to gain a sense of protein function, albeit at a lower level of confidence, *Ae. togoi* proteins that did not find an RBH match to the reference species’ proteome were annotated using a best hit (BH) BLAST approach. These *Ae. togoi* proteins were individually used as queries to search the reference species’ proteome again, taking the top-scoring match as the BH, with a minimum e-value threshold of 1e^−6^. This process was done separately for *Ae.* *aegypti, Ae. albopictus*, and *Ae. camptorhynchus* proteomes before the results were combined. Names for *Ae. togoi* protein transcripts were preferentially given based on the matching *Ae. camptorhynchus* annotation name, due to higher protein sequence identity matches in general, compared to *Ae.* *aegypti* and *Ae. albopictus* transcripts.

#### NHE3 protein sequence comparison

*Ae. togoi* NHE3 long and short isoform sequences were aligned using Clustal Omega.^123^ PKA phosphorylation binding domains were predicted using NetPhos (v3.1),^124^ reporting results that landed within gaps between the long and short NHE3 isoforms and had prediction scores greater than 0.5. Transmembrane domains were predicted using DeepTMHMM (v1.0.57).^125^

### Quantification and statistical analysis

#### Differential expression of whole larvae and dissected salt gland tissues

Transcript abundance from whole larval and dissected salt gland RNA datasets were separately measured using the Nextflow (v25.04.6) nf-core rnaseq pipeline (v3.22.2), which utilizes SALMON to generate count metrics for each transcript.^126^^,^^127^ Transcript counts were analyzed for differential expression with DESeq2 (v1.42.1).^128^ Lists of differentially expressed transcripts were filtered for significance at an adjusted *p*-value (padj) threshold of 0.05. Volcano plots were made using EnhancedVolcano (v1.20.0).^129^ Transcript counts are represented as transcript per million (TPM) values in annotation tables. A sub-list of 314 transcripts relevant to ion regulation and osmoregulation were hand-selected from highly conserved families of ion and water transporters and channels and proteins regulating ion and water flux across and between cells.^85^^,^^86^^,^^87^^,^^88^^,^^89^^,^^90^^,^^91^^,^^92^^,^^96^^,^^130^^,^^131^

#### GO term enrichment

Functional information for predicted protein transcripts were drawn from GO-term and PFAM domain annotations provided by eggnog-mapper (v2.1.12).^132^^,^^133^ Three enrichment analyses were conducted. The first was to identify GO-terms enriched among transcripts with higher expression in the salt gland than in the whole larvae. Transcripts more highly expressed in the salt gland transcriptome were defined as those with reliable expression in either the whole larvae or salt gland transcriptome datasets (TPM >1, in at least two replicates across treatments) and were among the top 50% with positive TPM-fold change when compared to transcripts expressed in the whole larvae, in the matching freshwater or saltwater condition (Table S5A). These were compared to a background of transcripts with reliable expression data in either the whole larvae or salt gland transcriptome datasets (Table S5B). The second analysis was to identify GO-terms enriched in the whole larvae when the animal is under saltwater conditions. Transcripts with significant differential expression in whole larvae (adjusted *p*-value <0.05) between SW and FW treatment groups (Table S7B) were compared to a background of all transcripts with reliable expression in the whole larvae dataset (TPM >1, in at least two replicates across treatments) (Table S7C). The third analysis was to identify GO-terms enriched in the salt gland when the animal is under saltwater conditions. Transcripts with significant differential expression in the salt gland (adjusted *p*-value <0.05) between SW and FW treatment groups (Table S9C) were compared to a background of all transcripts with reliable expression (TPM >1, in at least two of replicates across treatments) in the salt gland dataset (Table S9D). GO-term enrichment analyses were conducted with topGO (v2.54.0), using a weighted FDR <0.05 cutoff to classify significant enrichment.^134^

## Supplemental information

Document S1. Figures S1–S6

Table S1. Rockpool field data

Table S2. Sequencing read info Genomic long-read info, Hi-C read info, RNA-seq read info.

Table S3. Repeatmasker estimate of genome repeat content *Aedes togoi* RepeatMasker output showing types of repetitive elements estimated in the genome; Aedes aegypti RepeatMasker output showing types of repetitive elements estimated in the genome.

Table S4. Aedes togoi protein transcript annotations All *Aedes togoi* protein transcript annotations, All *Aedes togoi* protein transcript annotations with additional columns for mean expression in salt gland, Transcripts with the 10 highest mean expression in freshwater in salt gland, Transcripts with the 10 highest mean expression in saltwater in salt gland, Transcripts with the 10 greatest negative logFoldChange in saltwater vs. freshwater, in salt gland, Transcripts with the 10 greatest positive logFoldChange in saltwater vs. freshwater, in salt gland.

Table S5. Salt gland enriched transcripts: GO terms Target transcript list used in GO-term analysis of salt gland-enriched transcripts, Background transcript list used in GO-term analysis of salt gland-enriched transcripts, and GO-term results from GO-term analysis of salt gland-enriched transcripts.

Table S6. Differentially expressed transcripts in whole larvae and salt gland Significantly differentially expressed transcripts in whole larvae, saltwater vs. freshwater, in descending order by adjusted *p* value, Significantly up-regulated transcripts in whole larvae, saltwater vs. freshwater, in descending order by adjusted *p* value, Significantly down-regulated transcripts in whole larvae, saltwater vs. freshwater, in descending order by adjusted *p* value, Significantly differentially expressed transcripts in salt gland, saltwater vs. freshwater, in descending order by adjusted *p* value, Significantly up-regulated transcripts in salt gland, saltwater vs. freshwater, Significantly down-regulated transcripts in salt gland, saltwater vs. freshwater, in descending order by adjusted *p* value.

Table S7. Whole larvae differentially expressed transcripts GO-terms GO-term results from GO-term analysis of differentially expressed transcripts in whole larvae, saltwater vs. freshwater, Target transcript list used in GO-term analysis of differentially expressed transcripts in whole larvae, saltwater vs. freshwater, Background transcript list used in GO-term analysis of differentially expressed transcripts in whole larvae, saltwater vs. freshwater.

Table S8. Ion regulation and osmoregulation transcripts Curated list of ion regulation and osmoregulation transcripts; ion regulation and osmoregulation transcripts significantly differentially expressed in whole larvae, in descending order by log fold change; ion regulation and osmoregulation transcripts significantly differentially expressed in salt gland, in descending order by log fold change

Table S9. Salt gland differentially expressed transcripts GO-terms GO-term results from GO-term analysis of differentially expressed transcripts in saltwater vs. freshwater, in salt gland, GO-term results from GO-term analysis of transcripts up-regulated in saltwater vs. freshwater, in salt gland, Target transcript list used in GO-term analysis of transcripts up-regulated in saltwater vs. freshwater, in salt gland, Background transcript list used in GO-term analysis of transcripts up-regulated in saltwater vs. freshwater, in salt gland, GO-term results from GO-term analysis of transcripts down-regulated in saltwater vs. freshwater, in salt gland.

Table S10. Ion regulatory transcript bubbleplot values Expression of pump protein transcripts in salt gland, Expression of transporter protein transcripts in salt gland, Expression of paracellular junction protein transcripts in salt gland, Expression of channel protein transcripts in salt gland, Expression of water channel protein transcripts in salt gland.

Table S11. Aedes genome assemblies available on NCBI
