# Supplementary material for: Ion regulatory mechanisms supporting hypersaline tolerance in the coastal rock pool mosquito Aedes togoi
Source: iScience. 2026 Nov 7;29(11):118153. doi: 10.1016/j.isci.2026.118153 (PMC13769471; doi:10.1016/j.isci.2026.118153)
Supplement: Supplementary file 1 [file mmc1.pdf]

iScience, Volume *29*

Supplemental information

Ion regulatory mechanisms supporting hypersaline tolerance in the coastal rock pool mosquito *Aedes togoi*

Jonathan Chiang, Elissa Khodikian, Orna Phelan, Ana K. Parra, Daniel A.H. Peach, Andrea C. Durant, and Benjamin J. Matthews

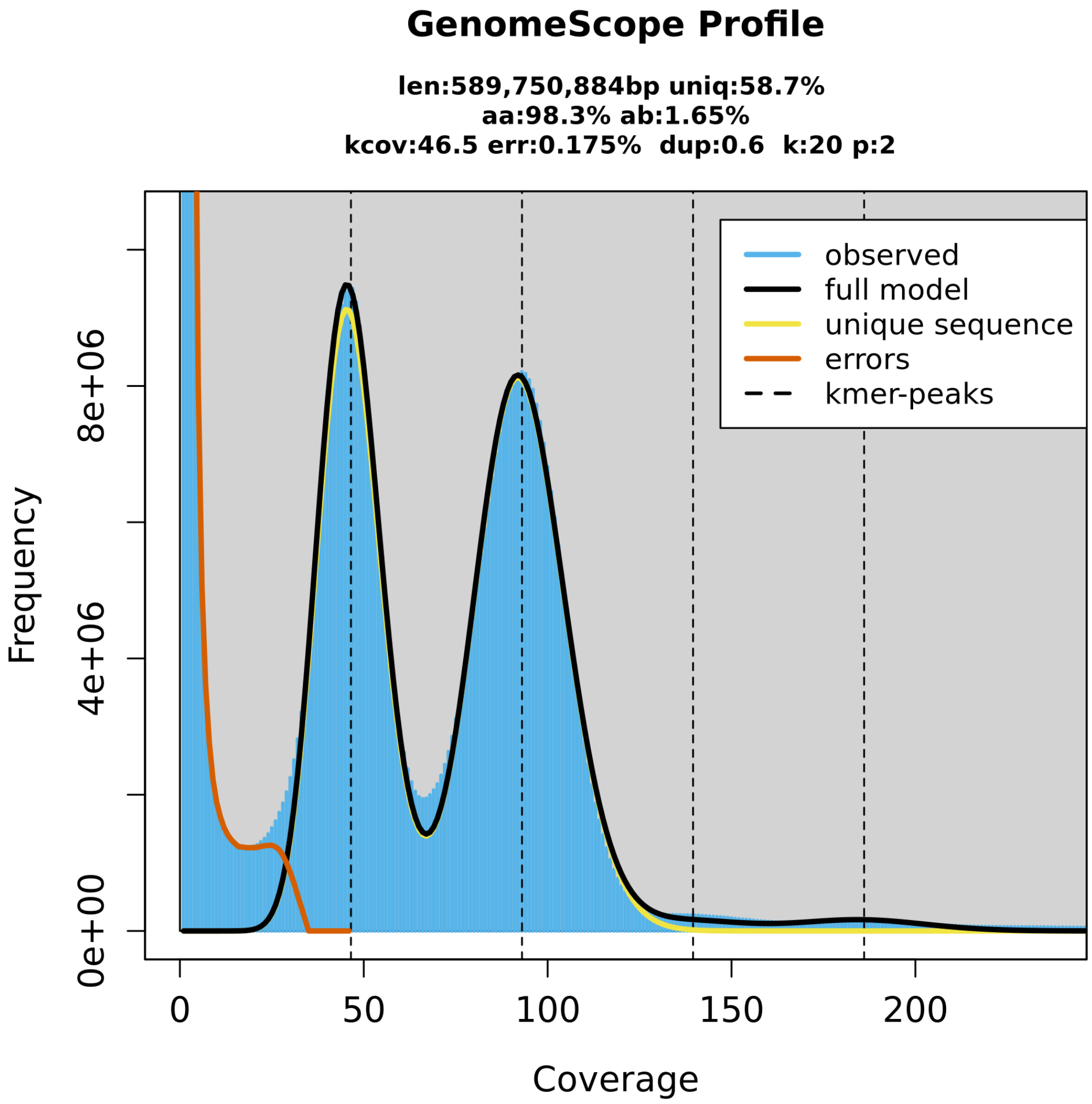

Figure S1. HiFi read k-mer-based estimate of genome characteristics
Ae. togoi genome characteristics were estimated based on our HiFi read k-mer counts. Genomescope2 was used to estimate genome length, heterozygosity, repeat content, and HiFi read sequencing error rate were estimated based on our deduplicated HiFi reads.

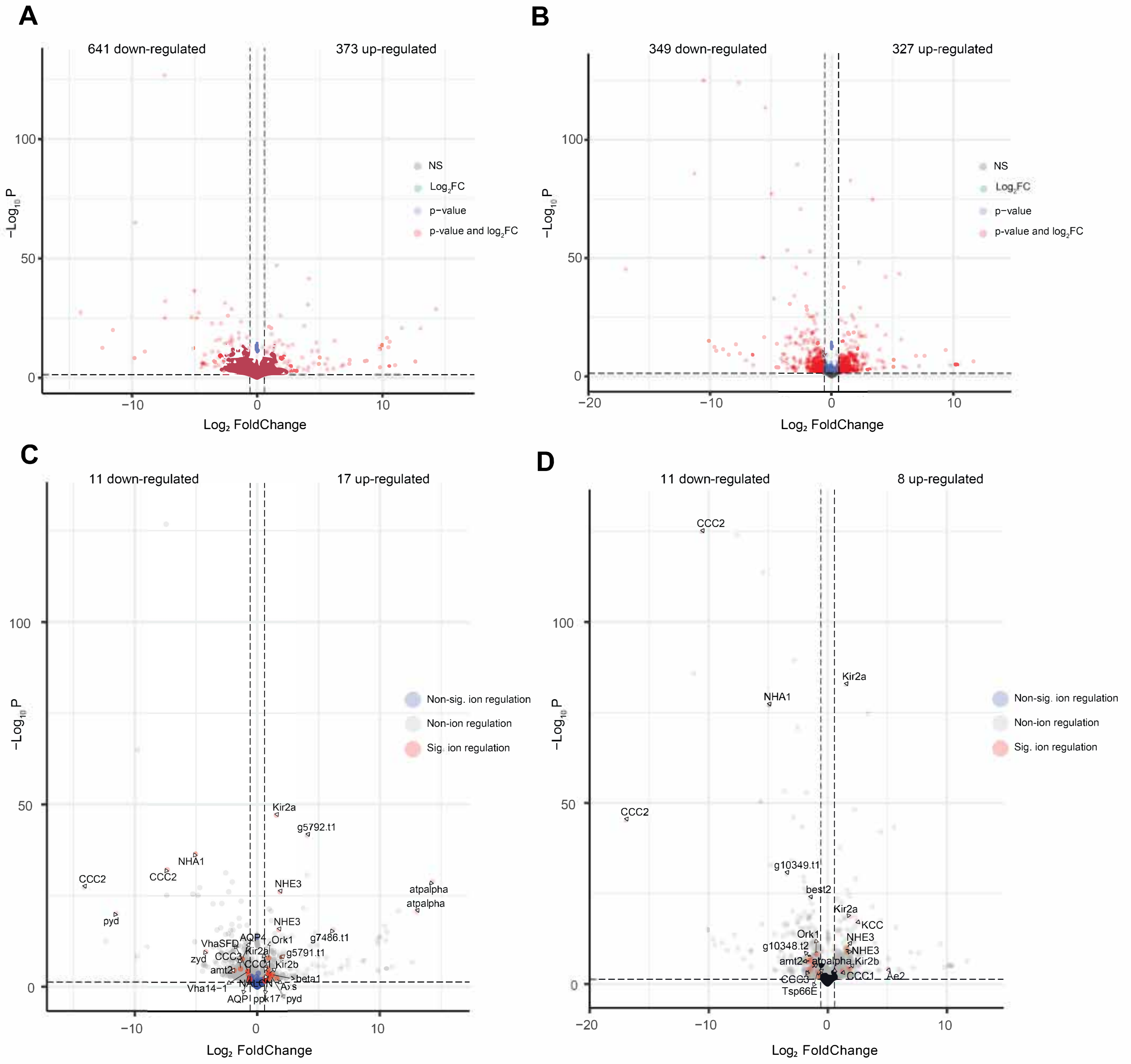

Figure S2. Differentially expressed transcripts between FW and SW conditions in whole larval and anal canal datasets. Differential expression between FW and SW conditions in (A) *Ae. togoi* whole larvae transcripts, (B) *Ae. togoi* larval anal canals transcripts, (C) ion regulatory transcripts from whole *Ae. togoi* larvae, and (D) ion regulatory transcripts from *Ae. togoi* larval anal canals. (A) 1,014 whole larval transcripts were differentially expressed out of 18,391 total transcripts. (B) 886 anal canal transcripts were differentially expresssed out of 18,391 total transcripts (C) 28 whole larval ion regulatory transcripts were differentially expressed out of 314 ion regulatory transcripts. (D) 19 anal canal ion regulatory transcripts were differentially expressed out of 314 ion regulatory transcripts. (A-D) Significance was determined by a threshold cutoff of p-value > 0.05 and $Log_2FoldChange > Log_2(1.5)$. Plots were generated with EnhancedVolcano.

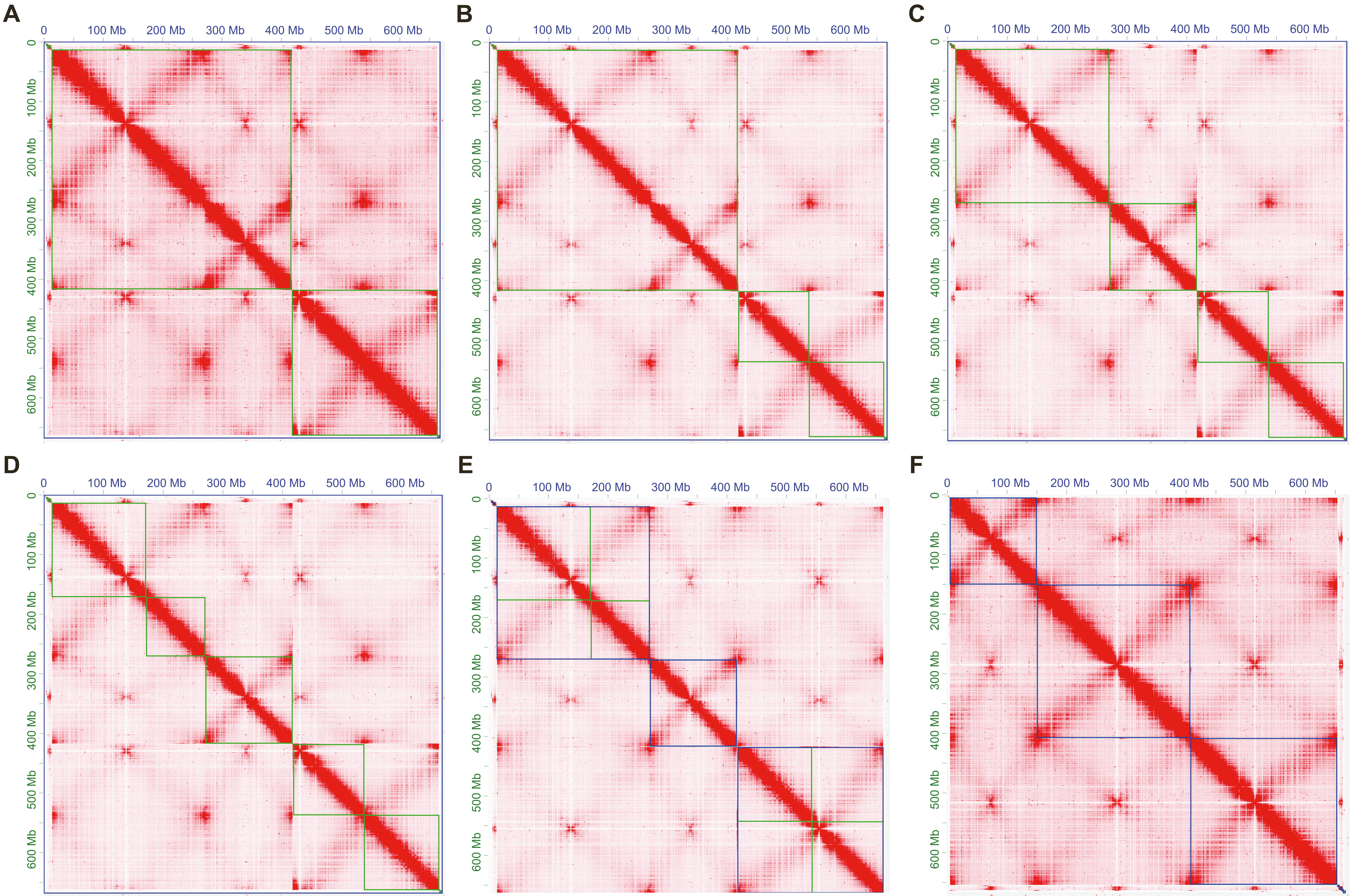

Figure S3. Manual curation process of *Aedes togoi* genome assembly in JuiceBox.
Edits to the scaffolded *Ae. togoi* assembly were implemented manually in JuiceBox, to fix points of mis-assembly and achieve correct chromosome structure guided by gene-order synteny to related *Aedes* species. (A-F) Hi-C contact maps showing Hi-C reads alignment signal (in red) mapped to the genome assembly, plotted against itself on both axes. Green boundaries represent scaffold/contigs while blue boundaries represent chromosome scaffolds. (A) Contact map showing the scaffolded *Ae. togoi* genome assembly, without any edits yet. (B) Contact map showing cut-site in the centromeric region of scaffold_2. (C) Contact map showing cut-site in scaffold_1, made to separate chromosomes 1 and 2. (D) Contact map showing cut-site in scaffold_1, made to separate mitochondrial genome sequence from the main assembly of nuclear genomic sequence. (E) Contact map showing signal patterning of assembly following swapping the arms of chromosome 3 (scaffold_2), revealing a more signal pattern more consistent with correctly assembled mosquito chromosomes. (F) Contact map showing the patterning of the curated *Ae. togoi* assembly, following processing in 3D-DNA's post-review pipeline.

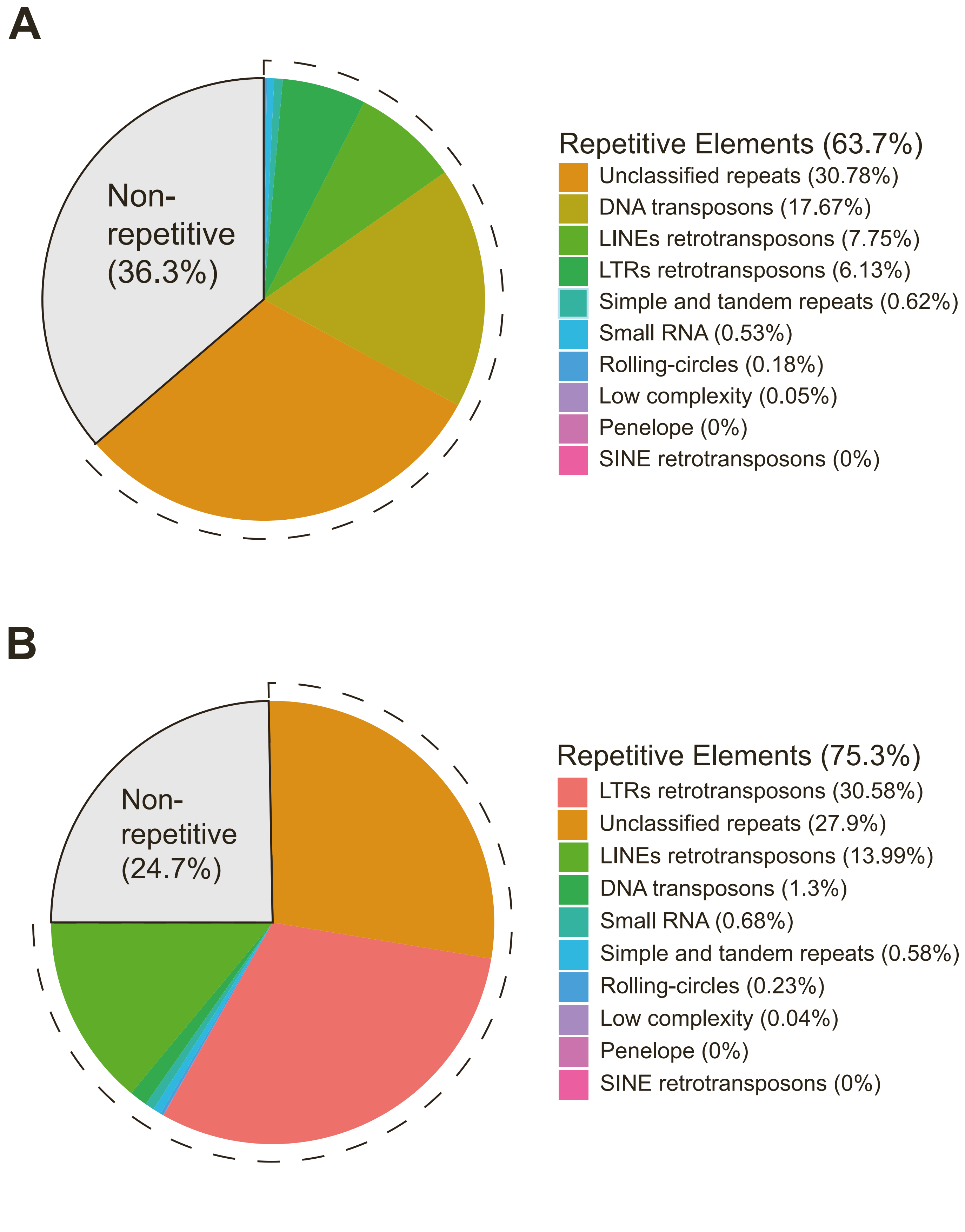

Figure S4. Repetitive element composition estimates by RepeatModeler and RepeatMasker.

(A) Repetitive element composition of *Aedes togoi* genome, using *de novo* repetitive element library predicted by RepeatModeler. Repetitive elements make up 63.7% of *Ae. togoi* genomic sequence.
(B) Repetitive element composition of *Aedes aegypti* genome, using the same methodology as a way to validate *Ae. togoi* repetitive element composition results. *Ae. aegypti* genome is estimated to be composed of 75.3% repetitive sequence, comparable to estimations in previous studies.

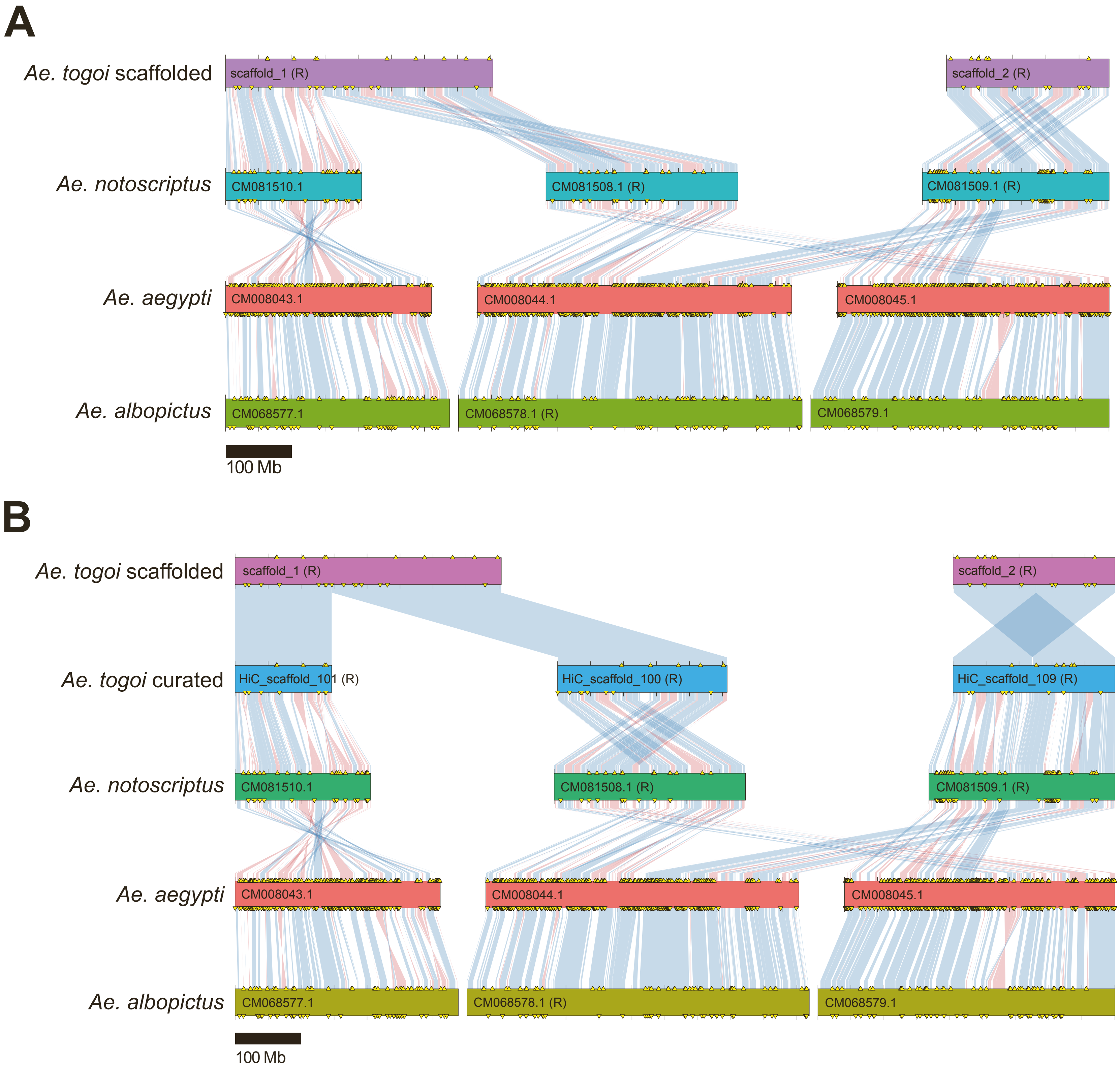

Figure S5. Chromosome structure of the scaffolded and curated *Aedes togoi* genome assemblies.
(A) Chromosomal synteny between the scaffolded (no manual curation) *Ae. togoi*, *Ae. notoscriptus*, *Ae. aegypti*, and *Ae. albopictus* genomes based on the location of orthologous BUSCO genes. (B) Chromosomal synteny between the curated *Ae. togoi*, *Ae. notoscriptus*, *Ae. aegypti*, and *Ae. albopictus* genomes, highlighting changes made to chromosome structure in curation process. Chromosomal inversions are highlighted in red while syntenic blocks with matching orientation are denoted in blue. Chromosomes with the majority of synteny on the opposite strand are labelled with '(R)'. Plots made with chromsyn.

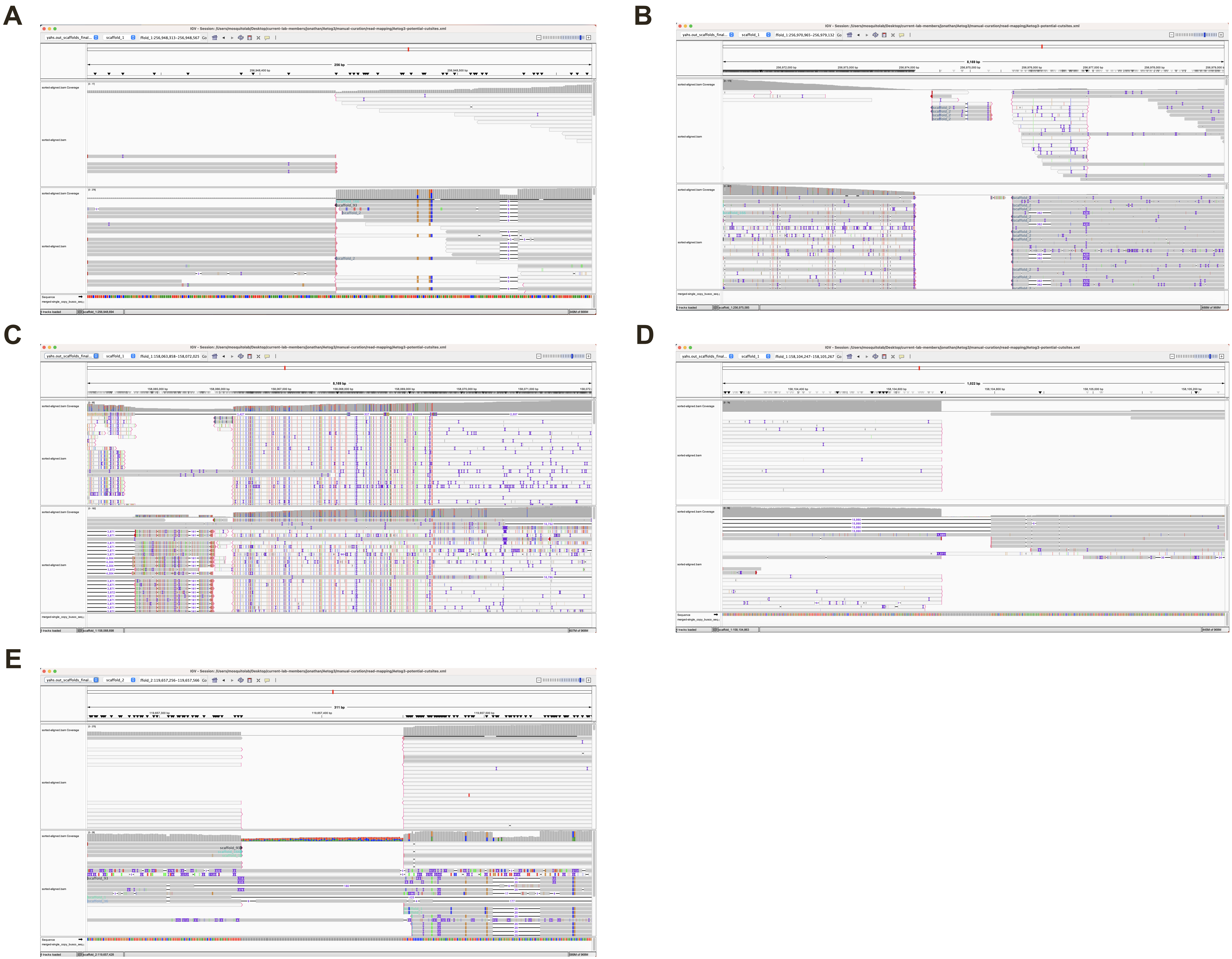

Figure S6. Cutsite selection in manual curation process of *Aedes togoi* genome assembly.
IGV was used to visualize long-read sequences aligned to the scaffolded *Ae. togoi* assembly, get a fine-resolution analysis of potential regions of misassembly, and identify suitable ‘cut-sites’ where the scaffold would be cut into fragments to be rearranged. Cut-sites were identified at regions where there was low read coverage or gaps in read alignment. (A,B) Regions around cuts (1.1 and 1.2) in scaffold_1 made to separate chromosomes 1 and 2. The region in-between these two cut-sites was discarded to debris as a separate contig due to having high repetitive content and no reads overlapping to either side of cut-sites. (C,D) Regions around cuts (3.1 and 3.2) in scaffold_1 made to excise mitochondrial genome sequence. The region in-between these two cut-sites was separated and removed as an intact assembly of the mitochondrial genome. (E) Region around cut (2.1) in scaffold_2 made to re-order the arms of chromosome 3.
